# Sexually dimorphic estrogen sensing in skeletal stem cells controls skeletal regeneration

**DOI:** 10.1038/s41467-022-34063-5

**Published:** 2022-10-30

**Authors:** Tom W. Andrew, Lauren S. Koepke, Yuting Wang, Michael Lopez, Holly Steininger, Danielle Struck, Tatiana Boyko, Thomas H. Ambrosi, Xinming Tong, Yuxi Sun, Gunsagar S. Gulati, Matthew P. Murphy, Owen Marecic, Ruth Tevlin, Katharina Schallmoser, Dirk Strunk, Jun Seita, Stuart B. Goodman, Fan Yang, Michael T. Longaker, George P. Yang, Charles K. F. Chan

**Affiliations:** 1 Department of Surgery, Stanford University School of Medicine, Stanford, CA 94305 USA; 2 Institute for Stem Cell Biology and Regenerative Medicine, Stanford University School of Medicine, Stanford, CA 94305 USA; 3 Department of Orthopaedic Surgery, Tongji Hospital, Tongji Medical College, Huazhong University of Science and Technology, Wuhan, China; 4 Department of Bioengineering, Stanford University, Palo Alto, CA 94305 USA; 5 Department of Surgery, University of Alabama at Birmingham, Birmingham, AL 35233 USA; 6 Birmingham VA Medical Center, Birmingham, AL 35233 USA; 7 Division of Plastic and Reconstructive Surgery, Stanford Hospital and Clinics, Palo Alto, CA USA; 8 Spinal Cord Injury and Tissue Regeneration Center Salzburg (SCI-TReCS), Department for Transfusion Medicine, Paracelsus Medical University of Salzburg, Salzburg, Austria; 9 Cell Therapy Institute, Paracelsus Medical University of Salzburg, Salzburg, Austria; 10 Center for Integrative Medical Sciences and Advanced Data Science Project, RIKEN, Tokyo, Japan; 11 Department of Orthopedic Surgery, Stanford University, Palo Alto, CA 94305 USA

**Keywords:** Mechanisms of disease, Ageing, Mesenchymal stem cells, Endocrinology, Bone

## Abstract

Sexually dimorphic tissues are formed by cells that are regulated by sex hormones. While a number of systemic hormones and transcription factors are known to regulate proliferation and differentiation of osteoblasts and osteoclasts, the mechanisms that determine sexually dimorphic differences in bone regeneration are unclear. To explore how sex hormones regulate bone regeneration, we compared bone fracture repair between adult male and female mice. We found that skeletal stem cell (SSC) mediated regeneration in female mice is dependent on estrogen signaling but SSCs from male mice do not exhibit similar estrogen responsiveness. Mechanistically, we found that estrogen acts directly on the SSC lineage in mice and humans by up-regulating multiple skeletogenic pathways and is necessary for the stem cell’s ability to self- renew and differentiate. Our results also suggest a clinically applicable strategy to accelerate bone healing using localized estrogen hormone therapy.

## Abstract (web summary)

How bone-related sexually dimorphic traits are regulated hasn’t been examined at the stem cell level. Here the authors show that skeletal stem cells (SSC), in female but not male mice, are directly controlled by estrogen signaling, which could be augmented to improve fracture repair.

## Introduction

Sexually dimorphic tissues are formed by cells that are regulated by sex hormones^1–4^. While a number of systemic hormones and transcription factors are known to regulate proliferation and differentiation of osteoblasts and osteoclasts, the mechanisms that determine sexually dimorphic differences in bone regeneration have been elusive^5–7^. Aging and age-related disease also show a marked sexual bias^8^. Previously, complex changes associated with aging were seen in many endocrine glandular tissues, resulting in dysregulation of steroid^9,10^ and amino acid-derived hormones^11,12^. Increasing evidence suggests that sex hormones, beyond their role in promoting sexual dimorphism, regulate stem cell self-renewal, differentiation, and proliferation^13–18^. Skeletal tissue exhibits sex differences in morphology and physiological function^19^. Clinically this is also manifested in differential epidemiology of osteoporosis and fracture risk^20,21^. In women, the risk of developing osteoporosis accelerates after menopause while incidence of the disease in men is progressive with age^22^. Current therapies of osteoporosis target fracture prophylaxis. However, due to non-union or disability up to a third of hip fracture patients can become totally dependent^23^, and the risk of institutionalization is great^24^. In a prospective study of 1133 patients with intracapsular fractures of the femoral neck, investigators found a significant increase in the incidence of non-union in females^25^. This raised the question of whether sex hormones, such as estrogen regulate skeletal stem cells in fracture regeneration.

In this study, to explore how sex hormones regulate bone regeneration, we analyzed bone fracture repair in young adult male, female, and female ovariectomized mice. We find the repair response to be significantly stronger in male mice is corresponding to a higher level of activated skeletal stem cell activity (SSC). We also find that the healing response in females to be dependent on ovarian function as ovariectomy nearly abolished activated SSC activity, leading to impaired skeletal regeneration and corresponding to a drop in systemic estrogen levels. These effects are mirrored clinically in postmenopausal women where lower rates of fracture healing are reported^20^. Mechanistically, we found that estrogen acts directly on the SSC lineage in mice and humans by up-regulating multiple skeletogenic pathways, and is necessary for the stem cell’s ability to self-renew and differentiate. Moreover, the response to estrogen also appears to be sexually dimorphic as SSCs from male mice do not exhibit similar estrogen responsiveness. Strikingly, SSC deficiency and bone loss following ovariectomy and aging can be reversed with a single, highly localized delivery of estrogen to the fracture site. Our results indicate that the osteoprotective actions of estrogen are mediated by direct SSC activation and suggest a clinically applicable strategy to accelerate bone healing using localized estrogen hormone therapy.

## Results

### Skeletal regeneration demonstrates sexual dimorphism

**Fig. 1:**
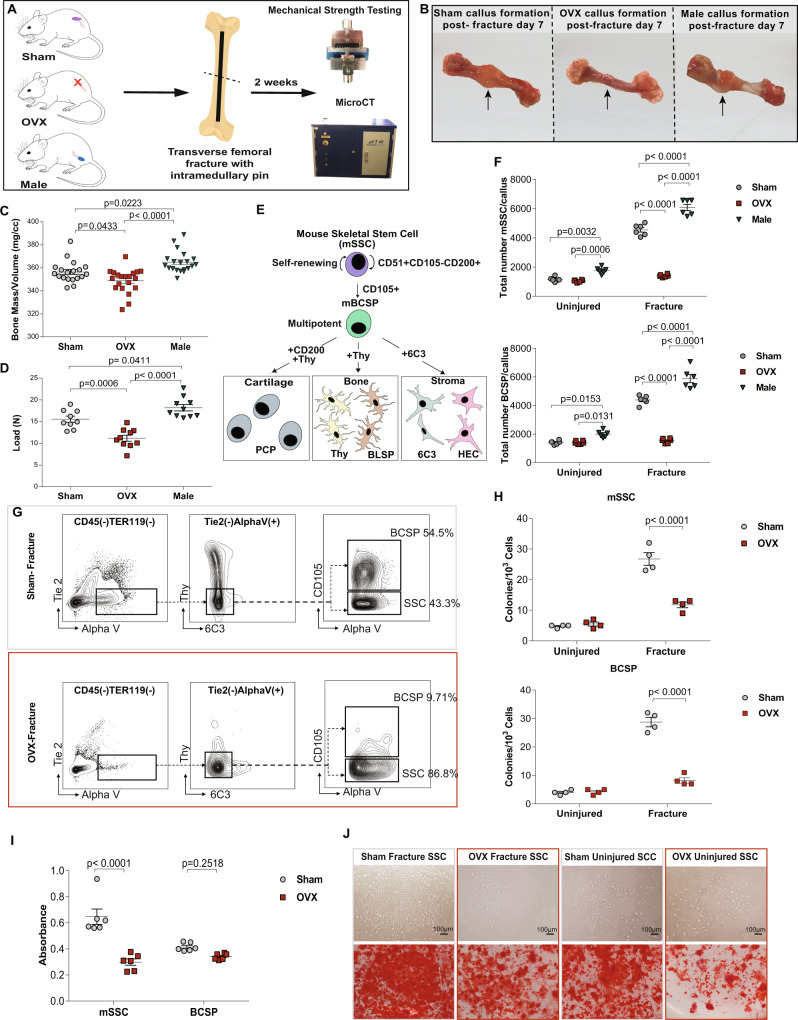
Skeletal regeneration demonstrates sexual dimorphism. **A** Schematic of fracture creation and assessment by MST and microCT. **B** Representative images of female sham (left), ovariectomy (center), and male (right) fracture callus at postfracture day 7. **C** Assessment of bone mass of trabecular bone in healing femora from sham vs OVX vs male mice (*n* = 20). **D** Maximal load to fracture (in newtons) of healing femora from sham (sham; grey) vs ovariectomized (OVX; red) vs male (male; green) mice (*n* = 10). **E** Schematic of mSSC lineage hierarchy: mSSC; pre-bone, cartilage, stromal progenitor (Pre-BCSP); mBCSP; Pro-chondrogenic cell (PCP); Thy+ osteogenic progenitor (Thy); B cell lymphocyte stromal progenitor (BLSP); 6C3 + stromal progenitor (6C3); hepatic leukemia factor-expressing cell (HEC). **F** Differences in absolute cell numbers of mSSCs (top) and mBCSPs (bottom) in sham vs OVX vs male mice (*n* = 5 per group), illustrating the decreased injury-induced expansion of OVX mSSCs and BCSPs when compared to sham mice. **G** FACS plots showing differences in the proportions of mSSCs and mBCSPs in post-fracture day 7 calluses from sham (top) vs OVX (bottom) mice. **H** Colony-forming assay of mSSCs (top) and mBCSPs (bottom) isolated at post-fracture day 7 from each experimental group. The total number of colonies were measured (*n* = 4). **I** Alizarin red staining spectroscopy shows an increased osteogenic potential of sham mSSCs and mBCSPs vs OVX mice (*n* = 6). **J** Representative images of colony size (*n* = 4) and alizarin red staining (*n* = 6) of uninjured and healing femora mSSCs from sham vs OVX mice. Bright-field microscopy, 10x. Scale bars 100μ m. The data in **C,D** are expressed as the means ± s.e.m;ordinary one-way ANOVA followed by Tukey’s multiple comparisons test; exact *P* value are indicated in the figures. The data in **F,H,I** are expressed as the means ± s.e.m; ordinary two-way ANOVA followed by Tukey’s multiple comparisons test (**F**) or Sidak’s multiple comparisons test (**H,I**); exact *P* value are indicated in the figures. For data and statistics, see Source Data File.

In this study, to determine whether sex hormones can affect fracture healing in mice we created pin-stabilized transverse femoral fractures in a well-established ovariectomized mouse model. Fracture healing was compared in adult ovariectomized mice (denoted as OVX), 14-week-old sham-operated female mice (denoted as sham), and male mice (denoted male), (Fig. 1A). OVX mice share multiple features and endocrine changes that are associated with human menopause^26–28^. We measured bone mass/volume in OVX mice and found that by 6 weeks after ovariectomy, there was a statistically significant decrease in bone mass compared to sham. In subsequent experiments, all OVX mice refer to mice 6 weeks after ovariectomy. It is well accepted that bone loss in OVX mice is the result of an imbalance of bone deposition and resorption leading to reduced skeletal mass and structural deterioration of skeletal tissue^5,29^. Enhanced osteoclast activity has previously been defined as the hallmark of osteoporosis in quiescent bone^27,30,31^. However, elimination of senescent osteoclast progenitors has no effect on age-related bone loss^32^. Despite this very little is known about the anabolic processes in bone integrity and fracture healing. At post-fracture week 2, macroscopic inspection of the dissected femora suggests a larger fracture callus in sham and male mice relative to OVX mice (Fig. 1B). Comparing male and sham female mice, we also observed a significant reduction in bone mass and mechanical strength in sham female calluses (Fig. 1C, D). Additionally, there is a significant difference in bone mass and mechanical strength in fracture calluses between male and OVX mice (Fig. 1C, D). These findings suggest that bone healing could be sexually dimorphic, and that skeletal regeneration is dependent on ovarian function in female mice. This is supported by the work of Manolagas’ group who have demonstrated that estrogens influence mammalian skeletal growth and maintain resulting in sexual dimorphism^33^.

Our laboratory has previously characterized the mouse skeletal stem cell (mSSC) lineage and its direct function in fracture repair (Fig. 1E)^34–37^. We showed that when mSSC were depleted by irradiation 12 h prior to fracture induction there was a significant reduction in callus formation and suppressed healing in the irradiated femora^34,36^. These findings are in agreement with other reports showing similar dependence of bone formation and healing in non-purified skeletal progenitors^38,39^. We therefore investigated whether impaired bone healing in OVX mice was due to a reduction in the number of mSSCs and downstream bone cartilage stroma progenitor cells (mBCSP). We have previously observed that the number of mSSCs is significantly increased during skeletal repair, and the injury-activated mBCSP forms a phenotypically distinct highly potent regenerative cell type in fracture calluses^34^. When we conducted FACS analysis of uninjured femora of OVX and sham mice 6 weeks after ovariectomy, when circulating estrogen levels have declined to ~20 pg/ml^40,41^, we found no significant difference in absolute cell numbers of mSSCs and mBCSPs (Fig. 1F). However the injury-activated expansion of mSSCs and mBCSPs was significantly reduced in postfracture day 7 calluses of OVX mice (Fig. 1F), in accordance with the peak point of callus formation and maximal skeletal progenitors^34–36^. Strikingly, the frequency of mSSC in both uninjured and injured femora of male mice was significantly higher than in femurs of female mice of the same age and weight. Furthermore, we observed a significant reduction in the relative expansion of mBCSPs compared to mSSCs in OVX mice (Fig. 1G). We next tested whether the difference in cellular proliferation and osteogenic differentiation contributed to the deficient stem and progenitor injury response. In vitro analyses showed that the number of colonies formed and osteogenic activity in injury-induced mSSCs and mBCSPs isolated from OVX mice were significantly reduced compared to sham controls (Fig. 1H–J). These results suggest that mSSC activity is differentially regulated in male vs female mice. In particular, the cell-extrinsic response of female mSSC to ovariectomized environment reduces clonogenicity and skeletogenic differentiation, and could provide a mechanism of impaired OVX bone healing.

### mSSC-mediated skeletal regeneration is estrogen dependent

**Fig. 2:**
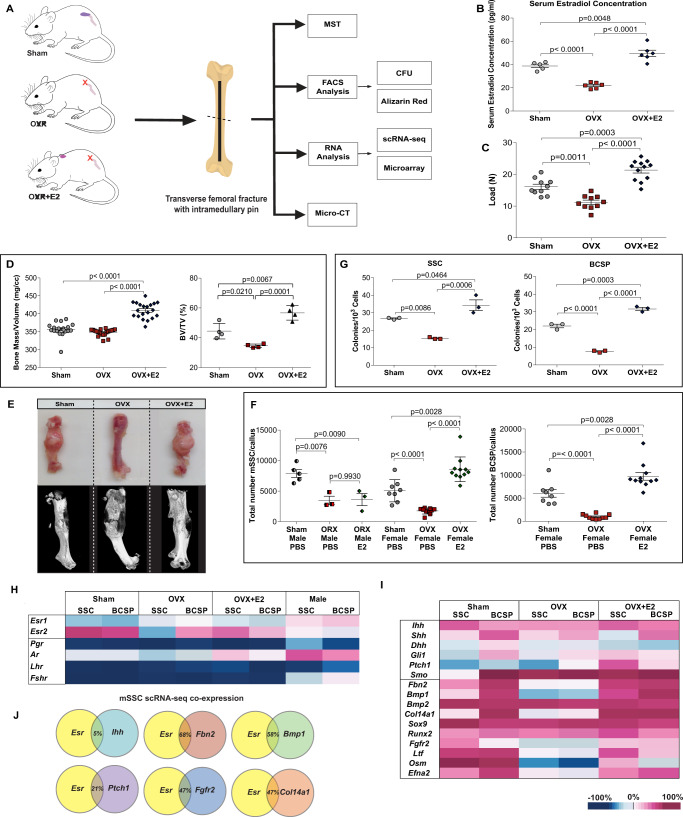
mSSC mediated skeletal regeneration is estrogen dependent. **A** Schematic of fracture creation on sham (sham), ovariectomized (OVX), and ovariectomized with 17-beta estradiol systemic rescue (OVX + E2) mice, and assessment by MST, microCT, FACS analysis, and RNA analysis. **B** Serum estradiol levels of sham vs OVX vs OVX + E2 demonstrates a significant decrease in serum estradiol levels in OVX vs sham. Elevated levels of estradiol were detected in OVX + E2 mice (*n* = 5). **C** Maximal load to fracture (in newtons) of healing femora from sham vs OVX vs OVX + E2 mice (*n* = 10). **D** Assessment of bone mass/volume of trabecular bone in healing femora from sham (grey) vs OVX (red) vs OVX + E2 (blue) mice (*n* = 20) and BV/TV of callus area from sham (grey) vs OVX (red) vs OVX + E2 (blue) mice (*n* = 4). **E** Representative photographs and radiographs of sham (left), OVX (center) and OVX + E2 (right) fracture callus at post-fracture day 7 demonstrating a larger callus in OVX + E2 mice and radiological non-union in the OVX mice. **F** Differences in absolute cell numbers of mSSCs (left) in sham vs ORX vs ORX + E2 for male mice (*n* = 3) and sham vs OVX vs OVX + E2 for female mice (*n* = 8), and mBCSPs(right) in sham vs OVX vs OVX + E2 mice (*n* = 8 per group). **G** Colony-forming assay of mSSCs (top) and mBCSPs (bottom) isolated at post-fracture day 7 from Sham, OVX, and OVX + E2 mice. There is significant expansion of mSSC and BCSP in systemic estradiol rescue compared to sham mice. The total number of colonies were measured (*n* = 3). **H** Heat map showing relative gene expression of sex hormone receptors in mSSCs and mBCSPs from fracture calluses of sham (left), OVX (center left), OVX + E2 (center right), and male (right) mice. **I** Heat map shows rescue of relative gene expression of mul-tiple skeletogenic factors (Ihh, Fbn2, BMP1, Col14a1, Fgfr2, Osm) in mSSCs and mBCSPs from fracture calluses of sham (left), OVX (center), and OVX + E2 (right) mice. Genes related to (top) Hh signaling; (bottom) skeletal development, growth and repair. Blue, low expression: red, high expression. **J** Single-cell RNA-se-quencing data demonstrates the percentage of co-expression of estrogen receptors and skeletogenic factors. The data in **B,C,D,F,G** are expressed as the means ± s.e.m; ordinary one-way ANOVA followed with Tukey’s multiple comparisons test; exact *P* value are indicated in the figures. For data and statistics, see Source Data File.

There are a number of hormones and growth factors released by the ovaries, including luteinizing hormone, progesterone and estrogen. The osteoprotective action of estrogen has been demonstrated in women and female rodents, but it has not been shown whether estrogen affects bone fracture healing at the stem cell level^42,43^. Estrogen primarily acts by regulating gene transcription via estrogen receptors 1 (ESR1, ERα) and 2 (ESR2, ERβ). We hypothesized that changes in mSSC-mediated skeletal regeneration following ovariectomy are estrogen-dependent. To test the role of estrogen in aberrant injury-induced bone regeneration of OVX mice, we treated OVX mice with continuous release 0.18 mg 17 β-estradiol subcutaneous pellets at the time of ovariectomy (OVX + E2) (Fig. 2A). With implantation of the 17 β-estradiol pellets, we restored serum estradiol levels in the OVX mice to a level even higher than those in sham mice (Fig. 2B). Microcomputed tomography (µCT) evaluation demonstrated increased bone mass density in OVX + E2 compared to sham controls (Fig. 2D). We further analyzed the fracture callus properties through bone volume/tissue volume µCT evaluation (Fig. 2D). We observed a significantly decreased BV/TV in OVX when compared to sham mice. Interestingly, we discovered an increased percentage of BV/TV in the OVX + E2 group compared to both OVX and sham groups. This is supported by our macroscopic findings. On inspection OVX + E2 femora had a visibly larger callus in contrast to sham and OVX mice (Fig. 2E). We then performed in-depth histological analysis by performing pentachrome staining and followed up with tissue composition analysis on both genders of mice (Supplementary Fig. 1A, B). We observed that sham male mice possessed denser bone structure with less marrow cavity when compared to female sham mice. This finding reflects the significant increase in bone mass/volume and bone volume/tissue volume percentage in male mice compared to female mice under µCT analysis at 2 weeks post-fracture. We also demonstrated that there are no significant differences in callus composition between sham male mice with or without E2 treatment. This further indicates that male mice do not benefit from E2 treatment in skeletal regeneration. In contrast, E2 treatment in female mice significantly increased the percentage of bone in callus of both sham and OVX female mice. These findings display sexual dimorphism in the estrogen response of skeletal stem cell regeneration on a histological, radiological and functional level. Additionally, mechanical strength testing (MST) of healing femora was significantly stronger in OVX + E2 mice (Fig. 2C). To determine whether this improvement was associated with changes in mSSC or mBCSP activity, we profiled the cellular composition of sham, OVX and OVX + E2 mice at post-fracture day 7 calluses using FACS. mSSC frequency has been shown to peak at post-fracture day 7^34^. We found that the absolute numbers of both mSSCs and mBCSPs increased significantly with systemic estradiol treatment (Fig. 2F). In order to demonstrate sex-specific estrogen sensing in skeletal stem cells we compared sham females (without ovariectomy), sham males (without orchiectomy), ovariectomized females with sham PBS rescue and estradiol rescue, as well as orchidectomized males with sham PBS rescue and estradiol rescue (Fig. 2F). There are significantly fewer SSCs in response to fracture injury in sham female mice when compared to sham male mice. Importantly, the number of mouse SSCs decreased significantly in response to fracture injury following ovariectomy and orchidectomy. Interestingly, estradiol is responsible for the rescue of resident SSCs in OVX mice, however estradiol makes no difference on the number of mouse SSCs following fracture in OVX mice. These experiments on orchidectomized adult male mice clearly demonstrate sex-specific regulation of SSC mediated bone repair following fracture.

We found that FACS-sorted OVX + E2 mSSCs formed significantly more colonies in vitro than mSSCs from sham mice (Fig. 2G). Collectively, these results indicate that replacement of estradiol in ovariectomized mice can restore mSSC activity in fracture healing, and estradiol levels above normal enhance skeletal regeneration beyond the natural physiological limits of WT control mice. We conclude that estradiol is sufficient in rescuing bone-healing defects of ovariectomized mice.

To identify molecular changes in skeletal stem cell signaling that could alter skeletal repair in OVX mice, we compared the transcriptomes of mSSCs and mBCSPs from sham, OVX and OVX + E2 mice using gene chip analysis of extracted mRNA (Fig. 2H, I). We used the Gene Expression Commons platform to normalize our microarray data against a reference of more than 11,939 mouse and 25,229 human publicly available array experiments and perform differential gene expression analysis^44^. We first note that expressions of *Esr1* and *Esr2* are differentially expressed in male vs female mSSC. While *Esr1* is expressed in low levels on both male and female SSCs, *Esr2* is expressed at high levels on female but not male mSSCs. In contrast, the androgen receptor is expressed in high levels in males rather than *Esr2* demonstrating that the difference in SSC activity in male mice is mainly driven by the androgens *(*Supplementary Fig. 2A*)*. It was recently reported that floxed deletion of *Esr2/ER-beta* expression in osteoprogenitors using a Prx1-Cre driver during early development results in marked *increase* of uninjured trabecular bone volume^45^. In contrast, we noted reduction in *Esr2* expression in OVX mice relative to sham and OVX + E2, which was associated with decreased bone mass and impaired skeletal stem cell activity (Fig. 2I). After we excluded the potential dimorphism effect of E2 treatment on periosteum Stem cells in both genders by FACs(Supplementary Fig. 2B, C), we further tested the role of estrogen-dependent SSC activity in-vivo using a genetic deletion of ESR2 in transgenic mice (Supplementary Fig. 2D, E). This demonstrates no change in SSC amplification in knockout mice when compared to WT sham female mice. Interestingly, SSC amplification of WT female mice was increased when exposed to estradiol, however the difference was not detected in genetic deletion of ESR2 in transgenic mice. Both our *Esr2* Knockout and previous Prx1-Cre/Floxed ESR2 findings are due to developmental compensation with upregulation of related genes following a gene knockout as a direct consequence of protein function loss^46^. The ovariectomized model may allow normal ESR dependent skeletal development while providing a more physiological comparison for post-developmental menopause associated skeletal changes.

In order to understand why estrogen receptor expression was suppressed specifically in the skeletal microenvironment of OVX mice we performed single-cell RNA sequencing (scRNA-seq). This allowed us to investigate gene differentiation and co-expression between estrogen receptors and skeletogenic factors. Corresponding to the bone healing deficiencies in OVX mice, we also observed that expression of several mediators of bone healing, including Indian Hedgehog (*Ihh*)^30^, Bone Morphogenetic Protein 1 (*Bmp1*)^47^, Fibrillin 2 (*Fbn2*)^48^, Fibroblast Growth Factor Receptor 2 (*Fgfr2*)^49^, and Oncostatin-M (*Osm*)^50^ were down-regulated in fracture calluses of OVX mice but restored to wild-type levels in OVX + E2 (Fig. 2I). Interestingly, Bmp1 and Tll1 are co-expressed in bone and are both responsible for the production of procollagen precursors into mature collagen monomers. Knockout of Bmp1 and Tll1 alleles produces a model of osteogenesis imperfecta^47^. Furthermore, single-cell RNA sequencing of mSSCs demonstrated co-expression of ER responsive skeletogenic signaling pathways and Esr (Fig. 2J). These results demonstrate that these genes are differentially regulated in SSCs and BCSPs. Coexpression at the single cell level demonstrates that multiple genes are involved, suggesting the effects of estrogen on SSC are complex and polygenic.

### Estrogen directly regulates SSC proliferation in mice and humans

**Fig. 3:**
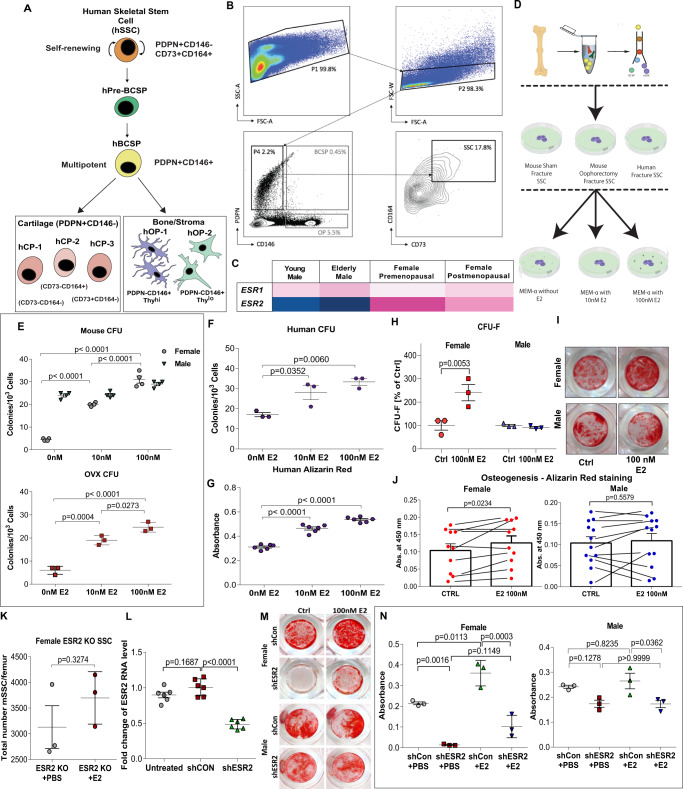
Estrogen directly regulates SSC proliferation in mice and humans. **A** Schematic of hSSC lineage hierarchy: hSSC; human pre-bone, cartilage, stromal progenitor (hPre-BCSP); hBCSP; human chondrogenic cell 1-3 (hCP); human osteogenic progenitor (hOP). **B** FACS gating scheme showing the identification of hSSCs. **C** Heat map showing relative gene expression of estro-gen receptors in hSSCs isolated from young male (far left), elderly male (center left), premenopausal female (center right), and postmenopausal female (far right) human specimens. **D** Schematic of estrogen concentration functional assays. SSCs were isolated from the fracture calluses from sham, ovariectomized mice, and postemenopausal female hip specimens. SSC were cultured in gradient concentrations of estradiol. The total number of colonies were measuredat day 14. **E** Colony-forming assay of mSSCs isolated at post-fracture day 7 from sham female and male mice (top) (*n* = 4), and OVX (bottom) mice cultured in gradients of estradiol (0 nM, 10 nM, and 100 nM) (*n* = 3). Cells were plated in biological and technical triplicates, and measured at day 14. **F** Colony-forming assay of hSSCs isolated from 8 postmenopausal female femoral head specimens obtained from hip arthroplasty. Cells were plated in biological and technical triplicates, and measured at day 14 (*n* = 3). **G** Alizarin red assay spectroscopy of hSSCs isolated from postmenopausal female femoral head specimens obtained from hip arthroplasty cultured in gradient concentrations of estradiol (0 nM, 10 nM, and 100 nM) (*n* = 6). **H** Colony-forming assay of hSSC isolated from 5 male and 4 female fracture specimens. Specific intrinsic difference between female and male fracture SSCs were grown in two different concentrations of estradiol (0 nM and 100 nM). Cells were plated in biological and technical triplicates and measured at day 14 (*n* = 3). **I** Representative images of Alizarin Red expression in male and female fracture specimens. **J** Alizarin red assay spectroscopy of hSSCs isolated from fracture specimens of both genders cultured in 0 nM and 100 nM estradiol. (*n* = 6). **K** ESR2 knockout mice do not respond to estradiol sensing (*n* = 3). **L** RT-qPCR analysis of ESR2 RNA expression level of human SSCs infected with lentivirus overexpressing scramble shRNA and ESR2 specific shRNA. (*n* = 3) **M** Representative image of Alizarin Red Staining of osteogenesis assay of hSSCs from both gender overexpressing scramble shRNA and ESR2 shRNA treated with PBS or 100 nM estradiol. **N** Quantification of Alizarin Red staining spectroscopy of hSSCs from both gender overexpressing scramble and ESR2 specific shRNA treated with PBS or 100 nM E2 (*n* = 3). The data in **E,F,G,H,L,N** are expressed as the means ± s.e.m; ordinary one-way ANOVA followed with Tukey’s multiple comparisons test; the data in J is expressed as the mean ± s.e.m, Paired t test; the data in K is expressed as the mean ± s.e.m, Unpaired t test; exact *P* value are indicated in the figures. For data and statistics, see Source Data File.

We next asked if estrogen receptor-dependent SSC activity is conserved in humans. To address this question, we examined highly purified human skeletal stem cell (hSSC) that our laboratory recently identified through prospective isolation^51^. Like mouse SSC, human SSC gives rise to bone, cartilage, and stroma, but not adipocytes, fibroblasts or muscle cells^41,43^ (Fig. 3A). However, hSSCs express a unique surface profile from mSSCs and are phenotypically CD235-Tie2-CD45-PDPN + CAD146-CD164 + CD73 + by immune profile. hSSCs of this phenotype are distinct from previously described human mesenchymal stems and bone marrow-derived stem cells that are often isolated by plastic adherence. Plastic adherent MSCs are often heterogenous populations consisting of multiple distinct subtypes of stem cells, the hSSC being one of these. The hSSCs we isolated exhibit true stem cell characteristics such as multilineage differentiation potential and self-renewal. They also generate downstream progenitors cells, such as the osteoprogenitor that possess only limited division cycles and restricted multi-lineage potential^51^. In this study, we isolated adult hSSCs from femoral heads after total hip arthroplasties as well as from fracture calluses collected from patients undergoing revision surgery. (Fig. 3B and Supplementary Table 1). The femoral head is of particular interest to differential estrogen sensing as the vast majority of hip fractures occur in post-menopausal women^29^. It is estimated that a post-menopausal woman has a 15-20% lifetime risk of hip fracture with only 30% of patients fully recovering^20^. Interestingly, serum estradiol levels are similar in postmenopausal women and adult men, 0-40 pg/mL^52–55^. However, despite similar estradiol levels women are at significantly greater risk of osteoporotic fracture than men, suggesting that sex differences in skeletal tissue may also involve differences in estrogen responsiveness^20,29,56^. In accordance with this possibility, we found that *Esr2* expression was also reduced in postmenopausal in women and men compared to premenopausal women (Fig. 3C). These trends parallel those observed in mSSCs isolated from OVX, sham, and male mice and implies that SSCs are capable of directly responding to changes in circulating E2 levels (Fig. 2H).

To confirm whether estrogen directly regulates mouse and human SSC activity, we performed functional assays on mice, and human SSCs isolated from postmenopausal female femoral heads in gradient concentrations of estradiol (0 nM, 10 nM, and 100 nM) (Fig. 3D). We found a dose-dependent increase in colony-forming units of mSSCs cultured in increasing E2 concentrations in females but surprisingly, not in male mice (Fig. 3E). We also found that E2 prompted an increase in colony forming units and osteogenic potential in hSSCs isolated from women but not men when cultured in increasing concentrations of estradiol (Fig. 3F–H). hSSCs isolated from fracture specimens in female patients increased their CFU levels by two folds in response to 100 nM E2 while hSSC from male patients were not responsive (Fig. 3H). We also determine that E2 increased the osteogenic response of hSSCs from women in vitro while hSSCs in men were not responsive (Fig. 3G, I–K). In order to understand the direct effects of estradiol in-vivo on human SSCs we generated qPCR data for ESR2 expression in the human SSCs transfected with lentivirus expressing control shRNA and lentivirus expressing ESR2 specific shRNA (Fig. 3L). This overcomes the problems with genetic compensation seen in KO murine models. We identified a significant reduction in ESR2 RNA levels in ESR2 specific shRNA human stem cells when compared to the untreated and shRNA control human stem cells. These findings are also reflected in osteogenic differentiation and function outcomes of human SSC in females and males (Fig. 3M, N). These results show that E2 could stimulate SSC activity directly but that in both mice and humans, the response is sexually dimorphic, with female SSCs significantly more responsive than male SSCs corresponding to increased expression of ESR2 in female SSCs.

### Localized delivery of 17-beta estradiol rescues mSSC expansion in skeletal regeneration

As previously described by Manolagas, estrogens influence the differentiation of osteoblast precursors^43^. Despite these important results, these studies were performed using downstream progenitors and not stem cells. This may reflect the differences from what we observed. In contrast we show that both mouse and human skeletal stem cells skeletal regeneration are estrogen dependent. We demonstrate that estrogen directly regulates mice and human bone proliferation on the stem cell level both in vitro and in vivo. Estradiol directly affects SSC/BCSPs without disturbing osteoclast function as we have demonstrated through TRAP staining. Furthermore, the effects of estrogen on osteoblast/osteoclast progenitor cells were demonstrated on KO mice by Manolagas et al.^57^. Our KO mice studies demonstrate no difference in survival and function on the stem cell level. This is because of genetic compensation seen, when there is upregulation of related genes following a gene knockout as a direct consequence of protein function loss^46^. To overcome this we performed shRNA studies which showed that knockdown of ESR2 in female SSC attenuated its osteogenesis ability and could be rescued with 100 nM estradiol treatments, which indicating that estradiol could stimulate SSCs activity directly in both mice and humans. This response is sexually dimorphic, with female SSCs significantly more responsive then males SSCs corresponding to increased expression of ESR2 in female SSCs.

**Fig. 4:**
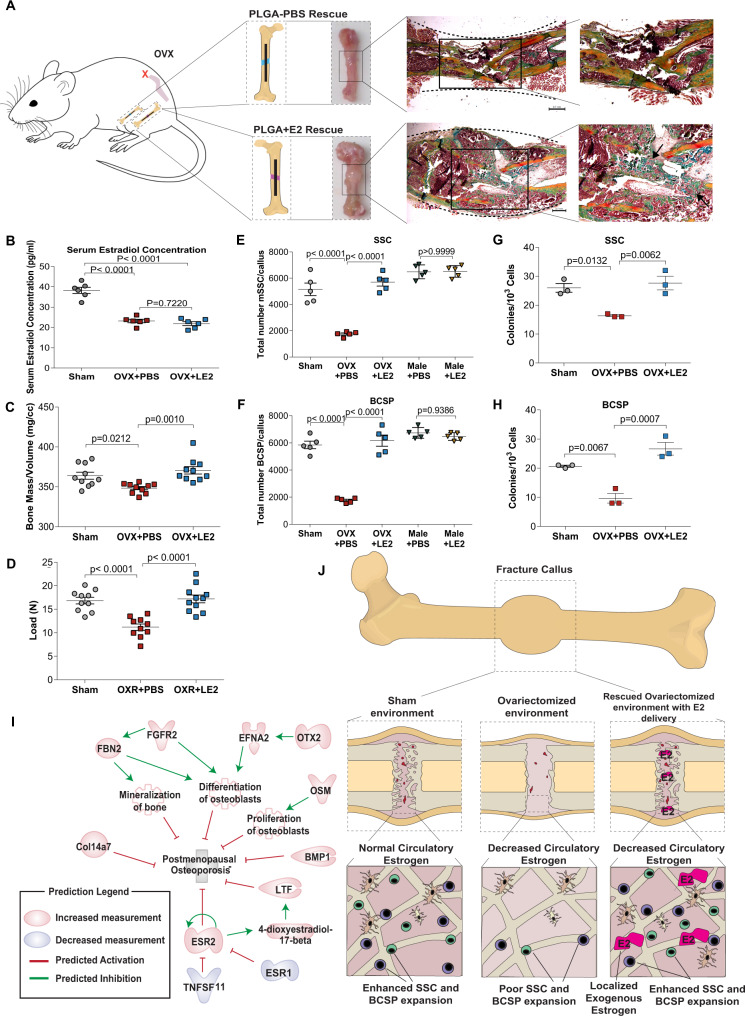
Localized delivery of 17-beta estradiol rescues SSC expansion in skeletal regeneration. **A** Schematic of unilateral localized delivery of 17-beta estradiol PLGA nanoparticle scaffold (OVX + LE2) and contralateral PBS PLGA nanoparticle scaffold (OVX + PBS). Representative images demonstrate a smaller callus in the OXR + PBS (top) compared to OVX + LE2 (bottom) fracture callus at post fracture day 7. Black arrows point to areas of endochondral ossification. **B** Serum estradiol levels of sham vs OVX vs OVX + LE2 mice demonstrate no increase in serum estradiol levels in the localized rescue group (*n* = 6). **C** Assessment of bone mineral density (BMD) of trabecular bone in healing femora from sham (grey) vs OVX (red) vs OVX + LE2 (blue) mice (*n* = 10). **D** Maximal load to fracture (in newtons) of healing femora from sham vs OVX vs OVX vs OVX + LE2 mice *(n* = 10)]. **E**, **F** Relative rescue of absolute cell numbers of mSSCs (**E**) and BCSPs **(F)** in sham (grey) vs OVX with sham local rescue (red) vs OVX with localized E2 mice (blue) (*n* = 5) (mSSCs and BCSPs). No relative rescue in male mice with localized E2 (yellow) compared to male with sham local rescue (green). **G**, **H** Colony-forming assay of mSSCs (**G**) and BCSPs (**H**) isolated at post-fracture day 7 from Sham, OVX, and OVX + LE2 mice. mSSCs and mBCSP were plated in biological and technical triplicates. **I** Schematic showing the effect of stimulation ESR2 on postmenopausal osteoporosis and the related protein interaction pathways. **J** Schematic showing the cellular and molecular mechanisms underlying impaired ovariectomized skeletal regeneration. Sham environment (left), OVX environment (center), and impaired fracture repair in OVX environment can be rescued by supplying localized E2 sensing (right). The data in B,C,D,E,F,G,H are expressed as the means ± s.e.m; ordinary two-way ANOVA followed by Tukey’s multiple comparisons test; exact *P* value are indicated in the figures. For data and statistics, see Source Data File.

Our findings clearly support the use of E2 hormonal therapy in osteoporotic treatment for women. However, we also recognize that systemic long-term exposure to estrogen is associated with greater risk of adverse results such as breast and endometrial cancer^58,59^, as well as venous thromboembolism^60^. Since our results show that SSCs are directly responsive to E2, and not through the secondary effects of E2 on systemic endocrine function, we asked whether highly localized, temporally regulated, delivery of E2 could improve fracture repair in an OVX setting. We applied a degradable PLGA nanoparticle scaffold coated with E2 or phosphate-buffered saline control to the defect sites of 14-week-old adult OVX and sham mice at the time of fracture fixation (Supplementary Fig. 3). Notably, visual inspection suggested larger callus size in localized estradiol-treated femora compared to the contralateral PBS-treated femora of the same mice demonstrating that local delivery of estradiol enhances bone healing. (Fig. 4A). Interestingly, serum estrogen levels were not affected by localized 17-β estradiol delivery when compared to OVX mice (Fig. 4B). MST and µCT analyses also revealed that localized E2-treated OVX fracture calluses (OVX + LE2) were significantly stronger and had significantly higher bone mass/volume when compared to PBS-treated controls corresponding to enhanced bone formation and as determined by histology (Fig. 4C–E). To determine whether this improvement was associated with changes in mSSC or mBCSP activity, we profiled the cellular composition of E2-treated and untreated post-fracture day 7 calluses using FACS and found that the absolute numbers of mSSCs and mBCSPs increased significantly in E2-treated calluses (Fig. 4F). Interestingly, localized delivery of estrogen failed to enhance injury-induced mSSC expansion in male mice (Fig. 4E**)**. We also found that FACS-sorted mSSCs isolated from estradiol-treated calluses of OVX mice formed significantly more colonies in vitro than mSSCs from OVX controls (Fig. 4G). To further elucidate the mechanism of estradiol effects on sham and OVX SSCs we performed differentiation analysis and Edu assays (Supplementary Fig. 4E–G). These findings directly demonstrate that estradiol increases the number of colonies by promoting the proliferation and differentiation of SSCs/BCSPs during injury, and not cell viability. These results indicate that local delivery of estradiol to the skeletal stem cell niche rescued OVX mSSC clonal activity in vivo, further emphasizing the importance of estradiol signaling to mSSC activity.We have also examined the effect of estradiol activity on mSSC differentiation into chondrocyte or adipocyte lineages. As we have noted previously, mSSC did not differentiate to adipocytes with or without estradiol^36^. We also did not see an effect of estradiol on chondrocyte differentiation in either male or female mSSC and mBCSP (Supplementary Fig. 5). Strikingly, we were able to demonstrate that localized delivery of estradiol also rescued mSSC and mBCSP deficiencies in fractures in 26 month-old aged female mice, which represent naturally post-menopausal mice (Supplementary Fig. 6). In order to address the role of estradiol on osteoclastic activity in bone healing we performed TRAP staining of callus isolated from injured female mice treated with PBS and estradiol (Supplementary Fig. 4A, B). We found that local delivery of estradiol had no effect on osteoclast activity within the fracture callus of the healing bone. Collectively, these findings demonstrate that targeted hormone therapy with localized estradiol could correct specific skeletal stem cell defects caused by natural or induced menopause, resulting in the restoration of mSSC-dependent repair in OVX mice and bypassing the potentially harmful effects of conventional estrogen replacement therapy (Fig. 4H, I).

In summary, our results provide new insight into the regulation of sexually dimorphic traits at the stem cell level during tissue regeneration. These findings corroborate numerous previous studies showing that mammalian tissue exhibits sexually dimorphic traits in the regeneration of various tissues, including haematopoietic^16^, vascular^61^ and somatic organs^62^. Here, we report that estrogen acts directly on female but not male SSCs in both mice and humans during bone regeneration. This led us to discover a method to directly modulate the skeletal niche signaling using temporal exposure to a localized delivery of PLGA nanoparticle and estrogen scaffold, which does not increase systemic levels of estrogen. We report successful treatment of impaired skeletal regeneration using this approach in a mouse model of post-menopausal osteoporosis. Thus, local stimulation of SSCs with estrogen loaded PLGA nanoparticles could be a readily applicable strategy to accelerate focal bone healing in women for a wide variety of clinical scenarios, from fracture healing, to dental implants. Focusing on SSCs and the SSC niche could reveal new possibilities for targeted combinatorial molecular therapy for skeletal and skeletal-related diseases. It was recently shown that estradiol increases hematopoietic stem cell (HSC) division, frequency, cellularity and erythropoiesis in the spleen^16,63^. As SSCs are capable of generating HSC niche cells, our new findings suggest a mechanism through which estradiol can also promote HSC activity through its effect on the niche. It will be interesting to see if recent findings on new pathways such as (Mg2/ CGRP) act synergistically with E2 and whether the effects are sexually dimorphic^64^. The co-delivery of these niche modulating factors would likely promote engraftment of transplanted SSCs, for instance to repair critical-sized defects and to facilitate hematopoietic engraftment^65,66^. Future efforts may identify hSSC-specific peptides that would enable systemic delivery of SSC-specific E2^66^.

## Methods

Our research complies with all relevant ethical regulations.Animals used in this paper were maintained in Stanford University Laboratory Animal Facility in accordance with Stanford Animal Care and Use Committee and National Institutes of Health guidelines. The study protocol was approved by Stanford’s Administrative Panel on Laboratory Animal Care. All procedures in this experiment were performed in accordance with the Stanford Administrative Panel on Laboratory Animal Care Protocol. The study protocol related to human tissue used in this paper is approved by the Institutional review boards(IRBs) at Stanford, which is also formally known as Administrative Panels for the Protection of Human Subjects. Fractured long bone specimens (20–90 years old) were obtained from Stanford Hospital in accordance with guidelines set by the Administrative Panels for the Protection of Human Subjects at Stanford (IRB-35711) and are considered exempt specimens per FDA guidelines 45 CFR 46.116 and therefore do not require consent forms as samples were considered biological waste.

### Experimental Procedures

The objective of this study was to understand the cellular and molecular mechanisms underpinning impaired fracture healing in osteoporosis.C57BL/6 mice were purchased from Charles River. The ESR2 Knock-out mouse strain, B6.129P2-Esr2tm1Unc/J were purchased from Jackson Lab. All mice were housed in temperatures of 65-75 °F with 40-60% humidity with a 14-hour light/10-hour dark cycle, and water is accessible at all times according to the protocols.The animals used in this experiment were 8 week old female mice. C57BL/6 aged female mice were operated on at 26 months old. 14 week old male were used in this study to account for the defined latent period in female mice bone mass/volume experiments (8 week old female mice + 6 weeks after ovariectomy). The study protocol was approved by Stanford’s Administrative Panel on Laboratory Animal Care. Male and female mice were age and weight matched. To avoid operator bias when dissecting the callus, we used the entire femur and standardized any confounders, including weight by normalizing the total number of SSCs and BCSP by including only the first 10,000 events on the FACS plot. For all experiments, the number of samples analyzed is outlined in the figure legends, and triplicates were performed for each experiment, unless otherwise stated. The experimental time points were previously optimized by our laboratory^34–36^. No outliers have been excluded from our analysis. Therapeutic treatment was randomization for each animal group.

### Ovariectomy

Eight-week-old mice were anesthetized with aerosolized isoflurane. Analgesia was administered and the surgical site was prepared. A lower back incision on each side was made. The uterine horn, oviduct and ovary with ovarian bursa were identified. The ovarian bursa was dissected away from the perinephric fat. The distal end of the uterine horn was ligated with 4-0 nylon and the distal portion was resected. The abdominal cavity was closed in two stages: the latissimus dorsi was closed with a running 4-0 vicryl followed by skin closure with interrupted 6-0 nylon. The latent period was defined as 6 weeks based on bone mass/volume experiments. Sham operations were performed in identical methods to the ovariectomy group, except ovarian structures were identified and not removed. Similarly, the abdominal cavity was closed in two stages: the latissimus dorsi was closed with a running 4-0 vicryl followed by skin closure with interrupted 6-0 nylon.

### Femoral fractures

Fourteen-week old mice were anesthetized with aerosolized isoflurane. Analgesia was administered, and the surgical site was prepared. A longitudinal incision was made in the skin parallel to the femur. The patella was dislocated laterally to expose the femoral condyles. The medullary cavity was reamed using a 25-gauge needle, before insertion of an intramedullary pin. A complete transverse, mid-diaphyseal fracture was made using microscissors. The pin remained in situ to stabilize the fracture to guide union. The patella was relocated, the muscles re-approximated, and the skin closed with 6-0 nylon sutures.

### Mechanical strength testing (MST)

MST was performed using a delaminator run by R.H. Dauskardt research laboratory at Stanford University. Fractured femur were harvested at postoperative Day 7. Mechanical load was a constant throughout all experiments. Samples were preloaded to 1 Newton (N) and underwent a three-point bend test at a compression rate of 1 micron/second. The maximum load (N) to fracture was recorded.

### PLGA fabrication and placement

The poly(D,L-lactide-co-glycolide) (PLGA, Sigma, MW 38-54 kDa) was dissolved in β-estradiol (Sigma) solution in chloroform. The concentration of PLGA was 15 w/v%. To cast the film, 150 µL β-estradiol containing PLGA solution was dropped on top of a teflon coated coverslip (diameter of 15 mm). The solvent was evaporated under ambient conditions for 6 h and then the formed film was further vacuum dried overnight. The obtained PLGA film was then punched into 4 mm diameter disks using a biopsy punch before use. The loading amount of β-estradiol per each disk was 5 µg. PBS-loaded PLGA scaffolds served as controls. For in vivo treatment, scaffolds were placed anteromedially on the defect and left in place until tissue harvest. In vitro kinetic assays were performed to show slow-release morphogen delivery. Each PLGA scaffold was maintained in 200 µl of PBS in a 96-well plate at 37 °C.

### Radiographic analysis

Radiographic analysis was performed at 7 day post fracture. Specimens were imaged using a calibrated X-ray micro-computed tomography unit (microXCT200, Carl Zeiss X-ray Microscopy, Inc.) at 4X magnification with a peak voltage of 40 kVp, an LE #2 source filter, and a beam-hardening constant of 2. Reconstruction analysis was performed using XMReconstructor software (version 8.2.3724). Bone mass/volume was determined using Avizo 9.0.0 post-processing software.

### Isolation of mouse skeletal progenitor cells

Fractured and uninjured femoral shafts were harvested at post-fracture day 7. Mouse skeletal progenitors were isolated as described by Gulati et al.^67^. In brief, fracture calluses were dissected and finely crushed using pestle and mortar. Tissues underwent serial enzymatic digestion in collagenase buffer [collagenase 2.2 mg/ml, deoxyribonuclease, IM CaCl2 (1000x), p188 (100X), 1 M HEPES (50X), Medium 199] at 37^o^C for 30 min under gentle agitation. Dissociated cells were filtered through a 40um nylon mesh and washed with FACS buffer [PBS, 2% fetal bovine serum, 1% pen strep, 1% p188]. Cells were pelleted at 200 G at 4^o^C and resuspended in FACS buffer. The suspension was layered onto a Histopaque gradient and centrifuged at 500 G for 15 min at room temperature with zero acceleration. The cloudy cellular interphase was aspirated, washed with FACS buffer, and centrifuged. The cells were stained with fluorochrome-conjugated antibodies against CD45 and Ter119 at dilution of 1:200(Biolegend, San Diego, CA, USA), Tie2 at dilution of 1:20 (eBioscience, San Diego, CA, USA), AlphaV integrin at dilution of 1:50 (eBioscience), CD105 at dilution of 1:50 (eBioscience), Thy1.1 at dilution of 1:100 (eBioscience), Thy 1.2 at dilution of 1: 100 (eBioscience), 6C3 at dilution of 1:100 (Biolegend), and CD200 at dilution of 1:50 (Biolegend) for fractionation by fluorescence-activated cell sorting (BD FACSAria II in the Shared FACS Facility in the Lokey Stem Cell Institute).

### Isolation of human bone progenitors

Femoral heads were obtained from young males, elderly males, premenopausal and postmenopausal females undergoing total hip arthroplasty. The bone marrow of the femoral head was isolated and human skeletal cells were separated from RBCs and bone dust by density gradient separation using 1:1 Histopaque 1.119 g/mL. The buffy coat was collected, washed with staining media, and the resulting cell suspension was depleted of CD45 + cells by magnetic-activated cell sorting (MACS). Cells were blocked with mouse IgG and stained with fluorochrome-conjugated antibodies against CD45 (Biolegend), CD235 (Biolegend), CD31(Invitrogen), CD202b (Biolegend), CD146 (Biolegend), Podoplanin (Invitrogen), CD164 (Biolegend), CD73 (Biolegend) at a dilution of 1:100. Flow cytometry was performed on FACS Aria II in the shared FACS Facility in the Lorry I. Lokey Stem Cell Institute. Gating schemes were established with fluorescence-minus-one (FMO) controls and 4’,6-diamidino-2-phenylindole (DAPI) was used for viability staining.

### Cell culture

FACS-isolated skeletal progenitor cells are primary expanded in MEM alpha medium with 10% fetal bovine serum and 1% penicillin-streptomycin (2% atmospheric oxygen, 7.5% CO_2_ conditions) for 3 days before counted and used for in vitro assay. For colony-forming units, one thousand cells of each population were plated in triplicate and maintained with MEM alpha medium with 10% fetal bovine serum and 1% penicillin-streptomycin (2% atmospheric oxygen, 7.5% CO_2_ conditions) for 2 weeks before colony counting. FACS-isolated human skeletal progenitor cells are primary expanded in MEM alpha medium with 10% human platelet lysate, 0.0002% heparin, and 1% penicillin-streptomycin (2% atmospheric oxygen, 7.5% CO_2_ conditions). For colony-forming units, one thousand cells of each population were plated in triplicate and maintained with MEM alpha medium with 10% human platelet lysate and 1% penicillin-streptomycin (2% atmospheric oxygen, 7.5% CO_2_ conditions) for 2 weeks before colony counting. For osteo-induction, cells were transferred to osteogenic differentiation media (MEM alpha medium, 10% fetal bovine serum or human platelet lysate, 100 mg/ml ascorbic acid, 10 mM b-glycerophosphate, and 100 nM dexamethasone) at 80% confluency. Alizarin red staining was performed after day-14. To quantify the alizarin red stain, each well was washed in PBS three times, then washed for 15 min in 20% methanol/ 10% methanol/ 10% acetic acid in ddH20 under gentle agitation to lift the stain. Absorbance was measured via spectrophotometry at 450 nm. Colony-forming units and alizarin red stain were also performed in various concentrations (0 nM, 10 nM and 100 nM) of 17-beta estradiol (Sigma). ImageJ 1.48 v was used for image processing.

### Transcriptional expression analyses

Microarray analysis was performed on highly purified, double-sorted SSC and BCSP populations. Each population was sorted into TRIzol (Life Technologies). RNA was extracted with the RNeasy Micro Kit (QIAGEN, Germantown, MD, USA) as per the manufacturer’s protocol. RNA was amplified twice with RiboAmp RNA amplification kit (Arcturus Engineering, Mountain View, CA, USA), streptavidin-labeled, fragmented, and hybridized to Affymetrix 430-2.0 arrays (Affymetrix, Santa Clara, CA, USA). Arrays were scanned with a Genechip Scanner 3000 (Affymetric) running GCOS 1.1.1. software and exported to DCHIP software for normalization. Raw microarray data were submitted to Gene Expression Commons (https://gexc.stanford.edu/)^37^, where data normalization was computed against the Common Reference, a large collection (*n* = 11,939) of publicly available microarray data from the National Center for Biotechnology Information Gene Expression Omnibus. Meta-analysis of the Common Reference also provides the dynamic range of each probe set for each gene. The probe set with the widest dynamic range was used for analysis. Heat maps representing fold change of gene expression were generated in Gene Expression Commons.

### Single-cell RNA sequencing

scRNA-seq was performed on a highly purified, double-sorted SSC population using FACS. Single cells were sorted into 96-well 4ul lysis plates composed of 0.2% Triton X-100 (Sigma), 2 U/μl of RNase inhibitor (Clontech), 2.5 mM dNTP (Invitrogen, 10297-018), 2.5 μM oligo dT30VN (IDT, custom: 5′-AAGCAGTGGTATCAACGCAGAGTACT30VN-3′), and 1:600,000 ERCC (External RNA Controls Consortium) ExFold RNA Spike-In Mix 2 (ERCC; Invitrogen, 4456739)) in nuclease-free water (Thermo Fisher Scientific, 10977023). Cells were spin down and plates kept at −80 °C until cDNA synthesis. cDNA synthesis from single-cell RNA was performed using oligo-dT primed reverse transcription with SMARTScribe reverse transcriptase (Clontech, 639538) and a locked-nucleic acid containing template-switching oligonucleotide (TSO; Exiqon, custom: 5′-AAGCAGTGGTATCAACGCAGAGTACATrGrG+G-3′). PCR amplification was conducted using KAPA HiFi HotStart ReadyMix (Kapa Biosystems, KK2602) with ISPCR primers (IDT, custom: 5′-AAGCAGTGGTATCAACGCAGAGT-3′). Amplified cDNA was then purified using 0.6× volume of Agencourt AMPure XP beads (Beckman Coulter, A63882). cDNA was quantified by high-sensitivity AATI 96-capillary fragment analyser (Advanced Analytical, Agilent HS NGS Fragment Kit (1–6,000 bp)). Concentration of wells containing single-cell cDNA was averaged to determine a dilution factor used to normalize each well to the desired concentration range (0.05–0.16 ng μl − 1). The content of four 96-well plates was then consolidated into 384-well plates without cherry-picking or removing wells not holding any single-cell cDNA. Subsequently the 384-well plates were used for library preparation (Nextera XT kit; Illumina, FC-131-1096) using a semi-automated pipeline. The barcoded libraries of each well were pooled, cleaned-up and size-selected using two rounds (0.35× and 0.75×) of Agencourt AMPure XP beads (Beckman Coulter), as recommended by the Nextera XT protocol (Illumina). Pooled libraries were sequenced on a NovaSeq6000 (Illumina) to obtain 2× 101-bp paired-end reads. scRNA-seq data were demultiplexed using bcl2fastq2 2.18 (Illumina). Raw reads were further processed using skewer v.0.2.2 for 3′ quality trimming, 3′ adaptor trimming, and removal of degenerate reads^68^. Trimmed reads were then mapped to the mouse genome v.M20 using STAR v.2.6.1plus^69^ (with an average of more than 70% of uniquely mapped reads), and counts per million (CPM) was calculated using RSEM v.1.3.1plus^70^. Data were explored and plots were generated using the Scanpy package (v.1.8.0.)^71^. Raw CPM values were mean- and log-normalized. Batch correction using Scanpy integrated ComBat(v3) was applied for cells derived from separate 96-well processing plates followed by data scaling.

### Copyright statement

There is no copyright infringement. The cartoons in this paper are generated by hand using Adobe illustrator.

### Statistical analysis

A two-tailed Student’s *t*-test was used for comparisons between 2 groups, an one-way analysis of variance (ANOVA) followed by Tukey’s multiple comparison test was used in the cases of unpaired comparison involving more than 2 groups. and an ordinary two-way ANOVA was used for comparison of matched data. Statistical analyses were performed using GraphPad Prism 9 (GraphPad). Deviation was graphically displayed as the standard error of the mean. Statistical significance was defined as *P* ≤ 0.05, exact *P* values were indicated in figures. All data points represent biological replicates, unless otherwise indicated in the figure legend.

### Reporting summary

Further information on research design is available in the Nature Research Reporting Summary linked to this article.

## Supplementary information

Supplementary Information

Reporting Summary

## Source data

Source Data

## Data Availability

All data are available from the corresponding author upon reasonable request. All single cell RNA-sequencing data and Microarray data generated in this study has been deposited at GEO under sample accession number GSE161477 and GSE213574. Source data is provided with this paper as a Source Data file. The remaining data are available within the Article or from the authors upon request. Source data are provided with this paper.
