# Author Correction: Sexually dimorphic estrogen sensing in skeletal stem cells controls skeletal regeneration

**DOI:** 10.1038/s41467-024-51829-1

**Published:** 2024-09-13

**Authors:** Tom W. Andrew, Lauren S. Koepke, Yuting Wang, Michael Lopez, Holly Steininger, Danielle Struck, Tatiana Boyko, Thomas H. Ambrosi, Xinming Tong, Yuxi Sun, Gunsagar S. Gulati, Matthew P. Murphy, Owen Marecic, Ruth Tevlin, Katharina Schallmoser, Dirk Strunk, Jun Seita, Stuart B. Goodman, Fan Yang, Michael T. Longaker, George P. Yang, Charles K. F. Chan

**Affiliations:** 1 Department of Surgery, Stanford University School of Medicine, Stanford, CA 94305 USA; 2 Institute for Stem Cell Biology and Regenerative Medicine, Stanford University School of Medicine, Stanford, CA 94305 USA; 3 Department of Orthopaedic Surgery, Tongji Hospital, Tongji Medical College, Huazhong University of Science and Technology, Wuhan, China; 4 Department of Bioengineering, Stanford University, Palo Alto, CA 94305 USA; 5 Department of Surgery, University of Alabama at Birmingham, Birmingham, AL 35233 USA; 6 Birmingham VA Medical Center, Birmingham, AL 35233 USA; 7 Division of Plastic and Reconstructive Surgery, Stanford Hospital and Clinics, Palo Alto, CA USA; 8 Spinal Cord Injury and Tissue Regeneration Center Salzburg (SCI-TReCS), Department for Transfusion Medicine, Paracelsus Medical University of Salzburg, Salzburg, Austria; 9 Cell Therapy Institute, Paracelsus Medical University of Salzburg, Salzburg, Austria; 10 Center for Integrative Medical Sciences and Advanced Data Science Project, RIKEN, Tokyo, Japan; 11 Department of Orthopedic Surgery, Stanford University, Palo Alto, CA 94305 USA

Correction to: *Nature Communications* 10.1038/s41467-022-34063-5, published online 30 October 2022

In this article the author name Ruth Tevlin was incorrectly written as Ruth Telvin. The original article has been corrected.

