# Supplementary material for: Sexually dimorphic estrogen sensing in skeletal stem cells controls skeletal regeneration
Source: Nat Commun. 2022 Oct 30;13:6491. doi: 10.1038/s41467-022-34063-5 (PMC9618571; doi:10.1038/s41467-022-34063-5)
Supplement: Supplementary file 1 — Supplementary Information [file 41467_2022_34063_MOESM1_ESM.pdf]

Supplemental Table 1. Human Specimen number, age, sex, and average hSSC per million FACS events

	Number of Specimens	Mean Total SSC/million events (+/-SD)	Sex	Age Range of Specimens
Human Femoral Head Specimens	8	771 (+/-509)	8F, 4M	62-29
Human Fracture Specimens	9	902 (+/-556)	4F, 5M	26-72

Supplementary Figure 1 Tissue composition analysis of callus from both genders of mice treated with E2

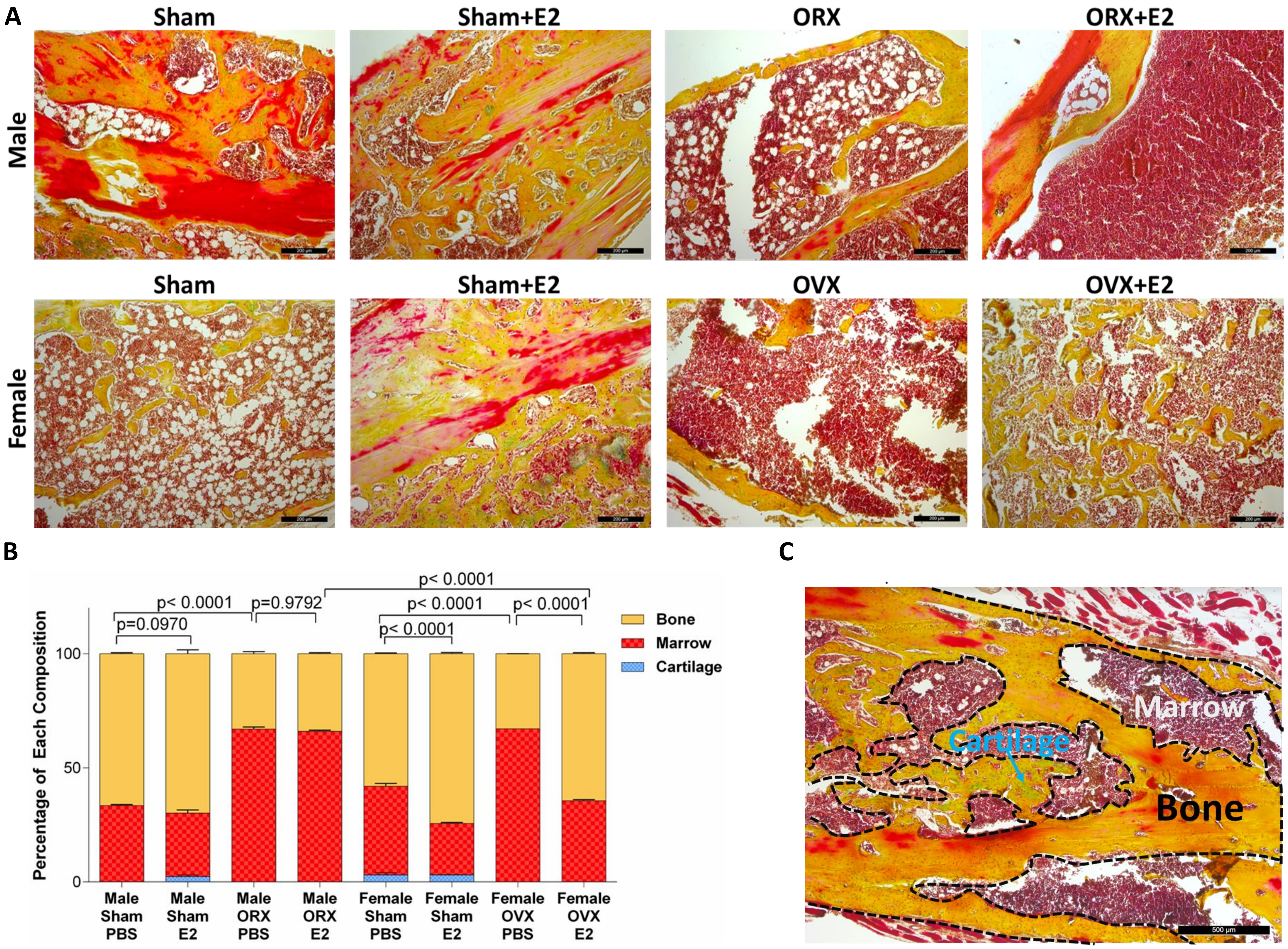

Supplementary Figure 1. Tissue composition analysis of callus from both genders of mice treated with E2
A). Representative image of Pentachrome staining of callus isolated from sham or ORX(male)/OVX(female) male and female mice treated with PBS or E2. Callus from OVX female mice regained dense bone structure under estradiol treatments, callus from ORX male did not. Bright-field microscopy, Scale bars 200µm. **B).** Quantitation of the tissue composition of the callus from the mice with the indicated treatments. This figure demonstrated E2 treatment promoted bone formation of the callus from both sham and OVX female mice, but has no significant effect in sham and ORX male mice in our injury model. Yellow signifies bone, red signifies bone marrow, and blue signifies cartilage within the callus (n=3 mice per group). **C).** Segmentation scheme showing the identification of bone, cartilage, and marrow area. The data in B are expressed as the mean$\pm$s.e.m; Percentage of Bone area are analyzed with Ordinary one-way ANOVA followed by Tukey's multiple comparison test; exact p value are indicated in the figure. For data and statistics, see source data files.

Supplementary Figure 2 The dimorphism effect of E2 is not observed in PSC or THY1+ osteoblast lineage

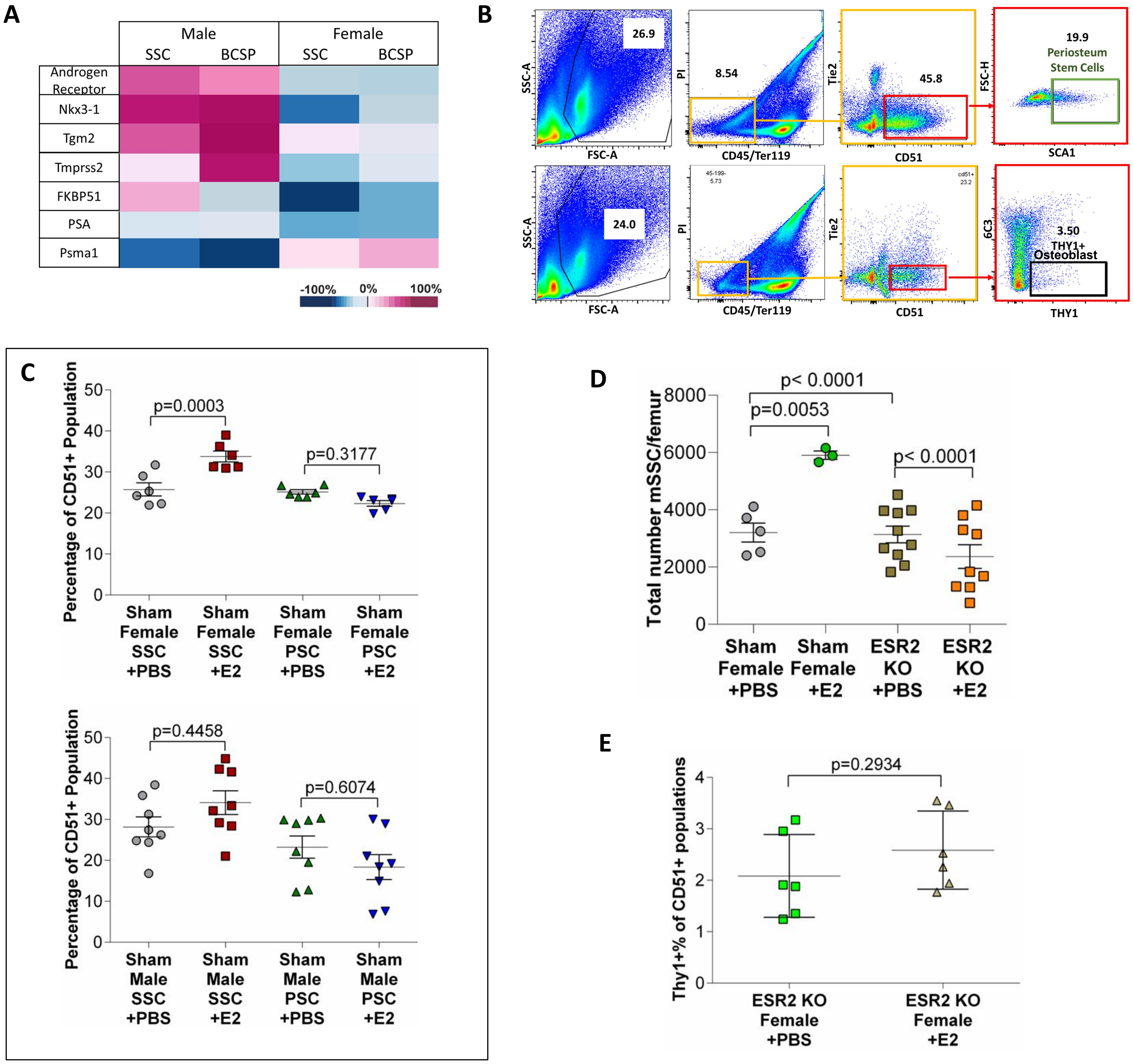

Supplementary Figure 2. The dimorphism effect of E2 is not observed in PSC or THY1+ osteoblast lineage

A). Heatmap showing relative gene expression of androgen receptor and well-known androgen receptor regulated target genes (NKX3-1, Tgm2, Tmprss2, Fkbp51, PSA, and Psma1) in mSSCs, mBCSPs isolated from adult male and female mice as previously described by the lineage hierarchy by Chan et al. (Chan 2015). The heatmap shows upregulation of gene expression of androgen receptor (AR) in male mice but not in female mice (red= high expression, blue = low expression). **B).** FACs gating scheme showing the identification of mouse Periosteum Stem Cells (PSC) and Thy1+ osteoblast lineage. **C).** FACs analysis in percentage of mPSCs and mSSCs in CD51+ cell population in injured sham male and female mice treated with PBS and E2. This analysis demonstrated E2 treatment has no effect on PSC in both gender but increased mSSCs numbers in callus from sham female mice. (n=8). **D).** FACs analysis in absolute cell number of mSSC in sham wild type female mice(n=3 per group) and ESR2 KO female mice(n=9 per group) treated with PBS and E2. This analysis demonstrated knockout of ESR2 blockaded increasing effect of E2 treatment on mSSC in callus from sham female mice. **E).** FACs analysis on Thy1+ osteoblast lineage percentage in CD51+ population in injured ESR2 KO female mice treated with PBS and estradiol. This analysis demonstrated the frequency of Thy+ osteoblast progenitors following ESR2 KO was not altered by E2 treatment. (n=6 per group). The data in C,D are expressed as the mean±s.e.m; Ordinary one-way ANOVA followed by Tukey's multiple comparison test; exact p value are indicated in the figure.The data in E are expressed as the mean±s.e.m; Unpaired t test; exact p value are indicated in the figure. For data and statistics, see source data files.

Supplementary Figure 3 In vitro release profile of E2 from PLGA

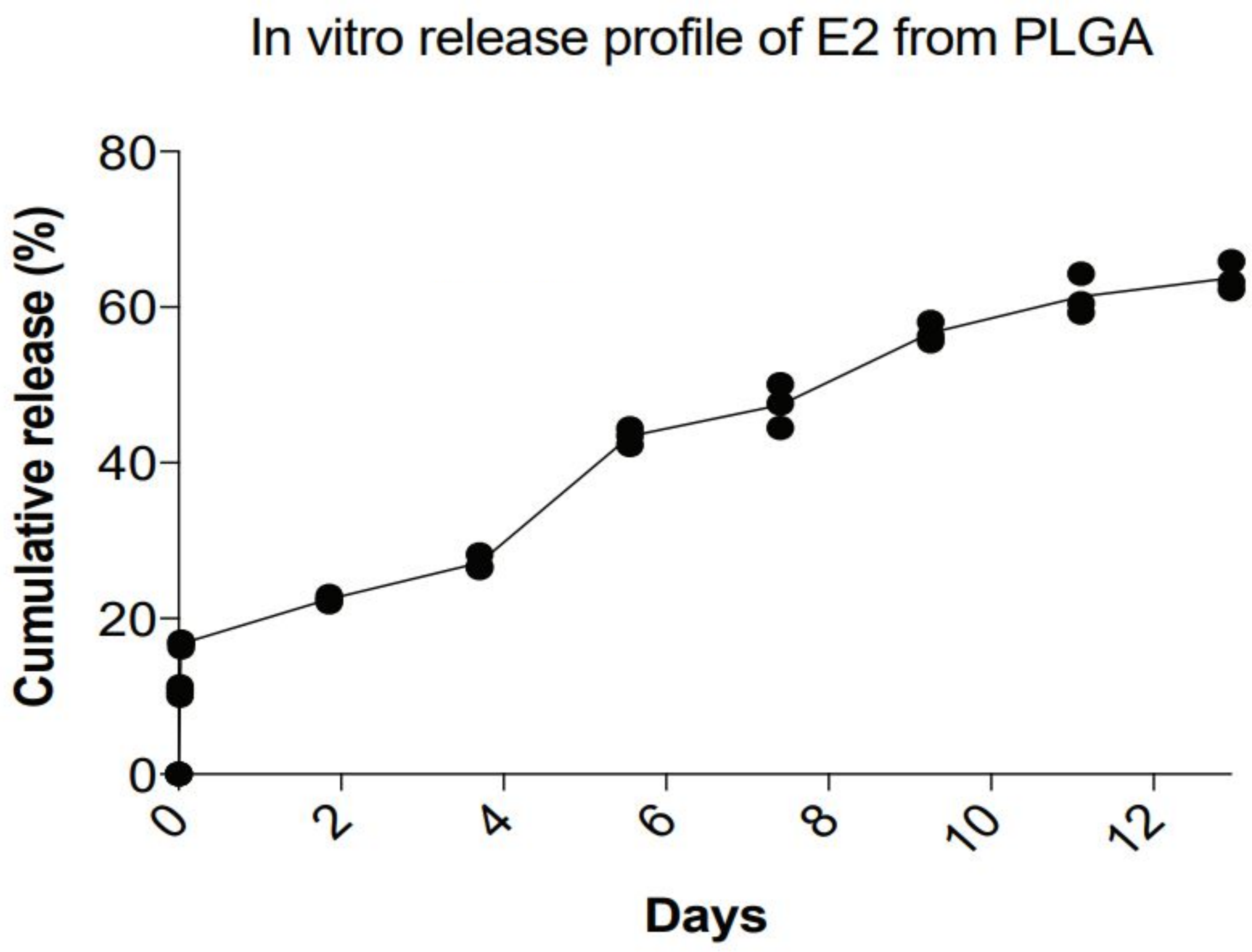

Supplementary Figure 3.In vitro release profile of E2 from PLGA
In vitro profile of estradiol from PLGA scaffold demonstrated sustained release over a 14 day period. The loading amount of E2 was 5ug. The concentration of PLGA was 15 w/v%. n=3.

Supplementary Figure 4. Estradiol promoted the proliferation and differentiation of SSCs/BCSPs during injury

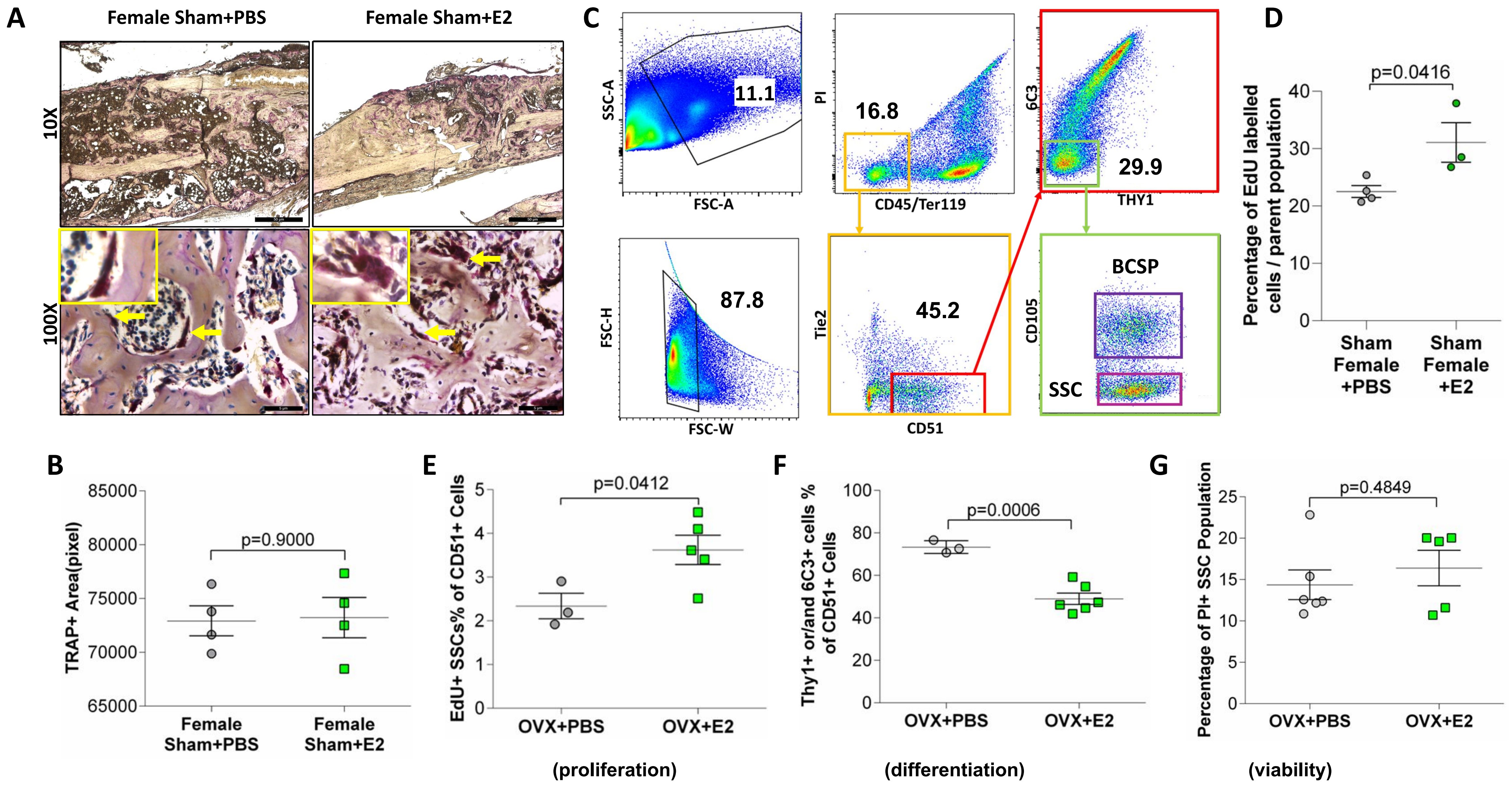

Supplementary Figure 4.Estradiol promoted the proliferation and differentiation of SSCs/BCSPs during injury
A). Representative image of tartrate-resistant acid phosphatase (TRAP) staining of callus osteoclast from Sham female fractured mice treated with PBS and E2 with arrows pointed at TRAP+ area. Bright-field microscopy, 10x. Scale bars 50μm, 100X. Scale bars 5μm(n=3) **B).** Quantification of TRAP+ area within callus under 50X demonstrated no difference in osteoclast activation level were detected between PBS and E2 treatment groups(n=3 per group). **C).** FACS gating scheme showing the identification of mSSCs and downstream bone cartilage stroma progenitor cells (mBCSPs). **D).** FACs analysis on percentage of EdU labeled mSSC/BCSP in sham+PBS(n=4) vs sham+E2 (n=3) female mice. This figure demonstrated estradiol treatment promoted colony expansion of SSC/BCSP in sham female mice. **E).** FACs analysis on percentage of Edu labeled mSSC within CD51 population in OVX+PBS (n=3) vs OVX+E2 (n=5) female mice. This analysis demonstrated E2 treatments promoted proliferation of mSSC in callus from OVX female mice. **F).** FACs analysis on percentage of Thy1+ or 6C3+ differentiated downstream lineage of mSSC/BCSP in CD51 population in OVX+PBS (n=3) vs OVX+E2 (n=5) female mice. This analysis demonstrated E2 treatments promoted maintenance of undifferentiated mSSC in callus from OVX Female mice. **G).** FACs analysis on percentage of PI+ cells in mSSCs population in OVX+PBS (n=3) vs OVX+E2 (n=5) female mice. This analysis demonstrated E2 treatments didn't influence cell viability of mSSC in callus from OVX female mice. The data in B,D,E,F,G are expressed as the mean$\pm$s.e.m; Unpaired t test; exact p value are indicated in the figure. For data and statistics, see source data files.
'

Supplementary Figure 5 The dimorphism effect of E2 is not observed in vitro chondrogenesis of mSSC and mBCSP

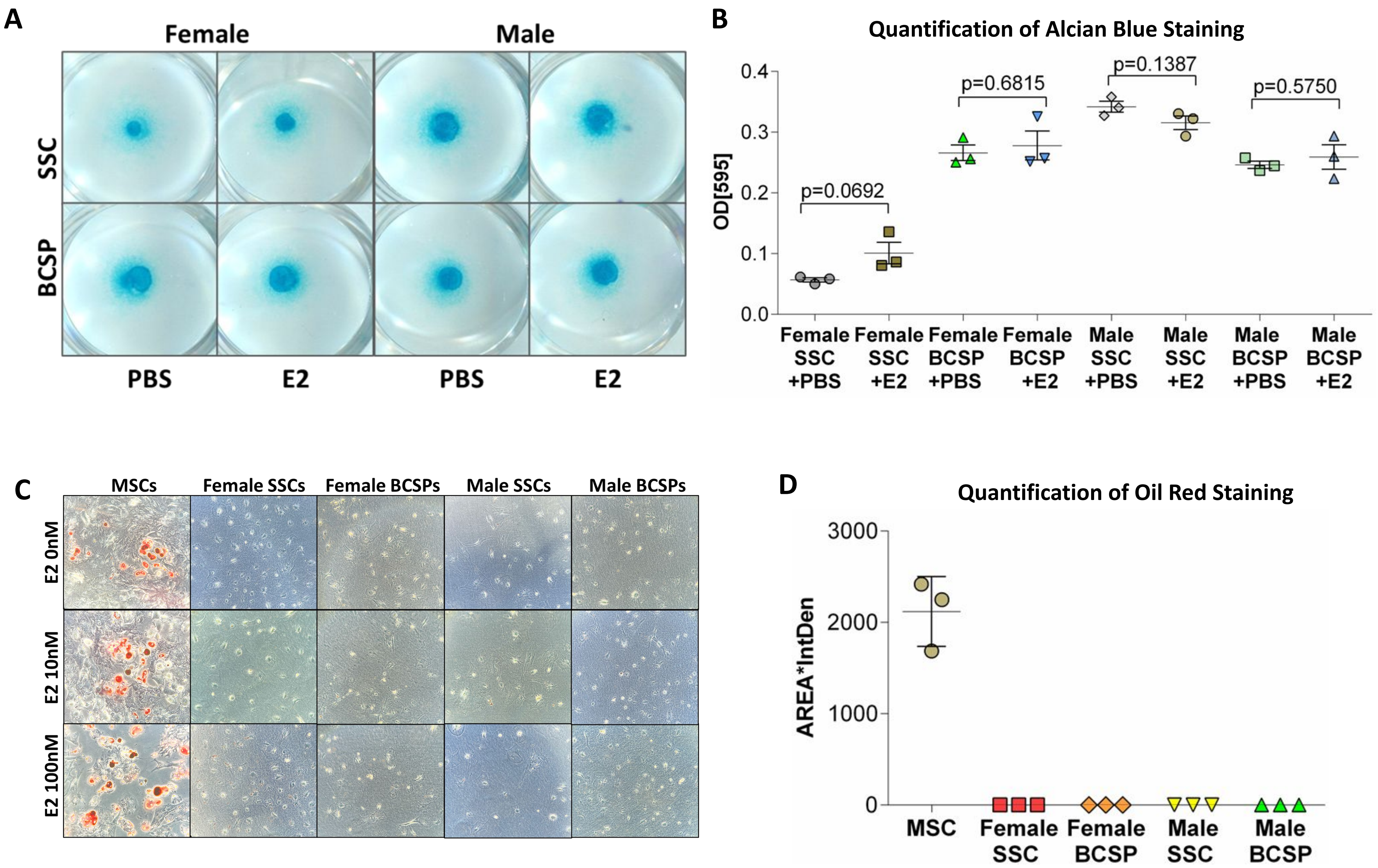

Supplementary Figure 5. The dimorphism effect of E2 is not observed in vitro chondrogenesis of mSSC and mBCSP
A). Representative image of Alcian blue staining of chondrogenesis assay in female and male mSSCs and mBCSPs treated with PBS and 100nM estradiol.(n=3) **B).** Quantification of Alcian blue staining of chondrogenesis assay in female and male mSSCs and mBCSPs with indicated treatments. This figure demonstrated E2 treatment has no effect on chondrogenesis of mSSC and BCSP (n = 3). **C).** Representative image of Oil-Red staining of adipogenesis assay of mice SSCs, and BCSPs from both gender treated with PBS , 10nM estradiol, and 100nM estradiol with pooled bone marrow mesenchymal stem cells(MSCs) from same mice as positive control(n = 3). **D).** Quantification of Oil-Red + Area of female and male mSSCs and mBCSPs treated with 10nM, 100nM E2, and PBS. This figure demonstrated mSSC and mBCSP has no potential in differentiating to adipose cells with or without E2 treatment. (n = 3). The data in B,D, are expressed as the mean$\pm$s.e.m; Unpaired t test; exact p value are indicated in the figure. For data and statistics, see source data files.

Supplementary Figure 6 localized E2 rescued mSSCs and BCSPs in naturally post-menopausal mice

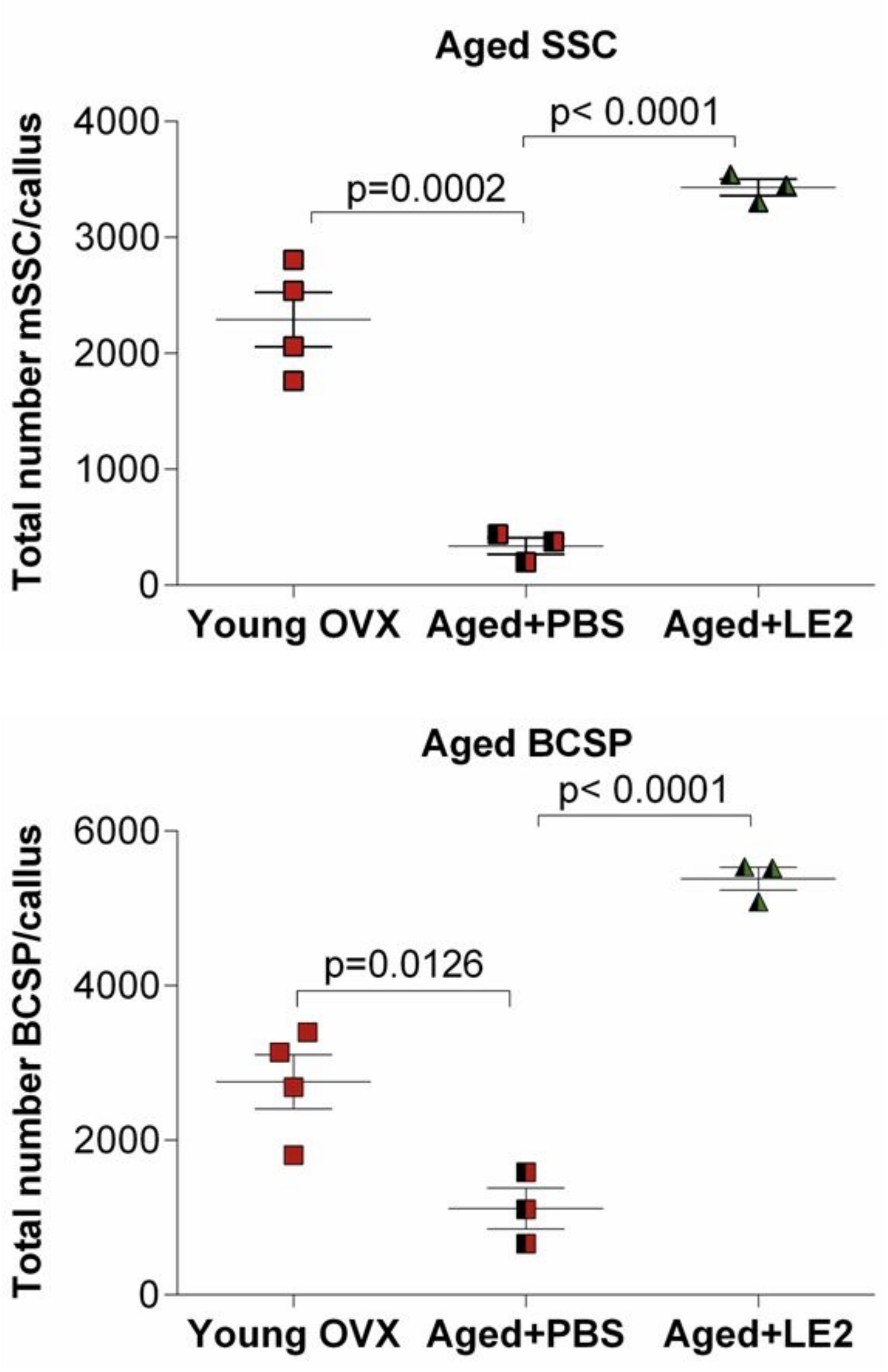

Supplementary Figure 6 localized E2 rescued mSSCs and BCSPs in naturally post-menopausal mice
Rescue of absolute cell number of mSSCs(top) and BCSPs (bottom) in Young ovariectomized mice vs 26 month aged with PBS controls vs 26 month aged with localized E2 (LE2) mice (n=3 per group). Data are expressed as the mean±s.e.m;Ordinary one-way ANOVA followed by Tukey's multiple comparison test; exact p value are indicated in the figure. For data and statistics, see source data files.
