# Supplementary material for: Sexually dimorphic estrogen sensing in skeletal stem cells controls skeletal regeneration
Source: Nat Commun. 2022 Oct 30;13:6491. doi: 10.1038/s41467-022-34063-5 (PMC9618571; doi:10.1038/s41467-022-34063-5)
Supplement: Supplementary file 2 — Reporting Summary [file 41467_2022_34063_MOESM2_ESM.pdf]

nature portfolio

Corresponding author(s): Charles KF Chan

Last updated by author(s): Sep 27, 2022

Reporting Summary

Nature Portfolio wishes to improve the reproducibility of the work that we publish. This form provides structure for consistency and transparency in reporting. For further information on Nature Portfolio policies, see our Editorial Policies and the Editorial Policy Checklist.

Statistics

For all statistical analyses, confirm that the following items are present in the figure legend, table legend, main text, or Methods section.

n/a	Confirmed	
☐	☒	The exact sample size (n) for each experimental group/condition, given as a discrete number and unit of measurement
☐	☒	A statement on whether measurements were taken from distinct samples or whether the same sample was measured repeatedly
☐	☒	The statistical test(s) used AND whether they are one- or two-sided<br>*Only common tests should be described solely by name; describe more complex techniques in the Methods section.*
☒	☐	A description of all covariates tested
☐	☒	A description of any assumptions or corrections, such as tests of normality and adjustment for multiple comparisons
☐	☒	A full description of the statistical parameters including central tendency (e.g. means) or other basic estimates (e.g. regression coefficient) AND variation (e.g. standard deviation) or associated estimates of uncertainty (e.g. confidence intervals)
☐	☒	For null hypothesis testing, the test statistic (e.g. F, t, r) with confidence intervals, effect sizes, degrees of freedom and P value noted<br>*Give P values as exact values whenever suitable.*
☒	☐	For Bayesian analysis, information on the choice of priors and Markov chain Monte Carlo settings
☒	☐	For hierarchical and complex designs, identification of the appropriate level for tests and full reporting of outcomes
☒	☐	Estimates of effect sizes (e.g. Cohen's d, Pearson's r), indicating how they were calculated

Our web collection on statistics for biologists contains articles on many of the points above.

Software and code

Policy information about availability of computer code

Data collection	Ruker Skyscan 1276 (Bruker Preclinical Imaging). Lago-X scanner (Spectral Instruments Imaging). A custom delaminator maintained by the R.H. Dauskardt laboratory (Stanford, CA). Flow cytometry was performed on FACS Aria II (BD Biosciences). Spectrometric measurements were conducted on a Ultraspec 2100 UV/Visible Spectrophotometer (Biochrom, Harvard Bioscience).
Data analysis	ImageJ 1.48v (NIH, http://imagej.nih.gov/ij/). CTAn and CTvox version 3.3.0r1383 software (Bruker). Software used for statistical analysis was Prism 9 GraphPad. FACS analysis was conducted using FlowJo v10 software. Raw microarray data were submitted to Gene Expression Commons (https://gexc.riken.jp) (Seita et al., 2012), where data normalization was computed against the Common Reference, which is a large collection of more than 11,939 mouse and 25,229 human array experiments deposited to the National Institutes of Health Gene Expression Omnibus (NIH GEO) database. For processing single cell RNA-sequencing data skewer11 for 3′ quality-trimming, 3′ adaptor-trimming, and removal of degenerate reads was used. Trimmed reads were then mapped to the mouse genome m38 using STAR 2.6.1, and counts per million (CPM) was calculated using RSEM 1.3.1. Scanpy integrated ComBat (V3) was used for batch correction.

For manuscripts utilizing custom algorithms or software that are central to the research but not yet described in published literature, software must be made available to editors and reviewers. We strongly encourage code deposition in a community repository (e.g. GitHub). See the Nature Portfolio guidelines for submitting code & software for further information.

Data

Policy information about availability of data

All manuscripts must include a data availability statement. This statement should provide the following information, where applicable:

- Accession codes, unique identifiers, or web links for publicly available datasets
- A description of any restrictions on data availability
- For clinical datasets or third party data, please ensure that the statement adheres to our policy

All data are available from the corresponding author upon reasonable request. All single cell RNA-sequencing data and Microarray data generated in this study has been deposited at GEO under sample accession number GSE161477[https://www.ncbi.nlm.nih.gov/geo/query/acc.cgi?acc=GSE161477] and GSE213574 [https://www.ncbi.nlm.nih.gov/geo/query/acc.cgi?acc=GSE213574]. Source data is provided with this paper as a Source Data file. The remaining data are available within the Article or from the authors upon request. Source data are provided with this paper.

Field-specific reporting

Please select the one below that is the best fit for your research. If you are not sure, read the appropriate sections before making your selection.

[x] Life sciences   [ ] Behavioural & social sciences   [ ] Ecological, evolutionary & environmental sciences

For a reference copy of the document with all sections, see nature.com/documents/nr-reporting-summary-flat.pdf

Life sciences study design

All studies must disclose on these points even when the disclosure is negative.

Sample size	The sample size for the number of replicates (n) for each experiment is provided in the figure legends. All sample size for each group in this paper are above the sample size required by ANOVA analysis (n = DF/k + 1; k = number of groups) with degrees of freedom (DF) set as 10.
Data exclusions	Animals that unexpectedly became morbid during the course of the experiment were excluded. During processing of single cell genomic data filtering was applied as described.
Replication	All data presented are biological replicates. All findings were reproduced at least 3 times. The only exceptions were incidents where animals died prematurely or unexpectedly before the requisite time point for analysis.
Randomization	Animals were allocated randomly into the different experimental groups. To obtain sufficient numbers, cells pooled from 6-10 mice were used for each replicate in in vitro studies.
Blinding	Assumed blinding were applied in the study. Investigators do not know the correspondence between group and treatment before the end of the study. Investigators were not blinded during data collection and analysis. Controls and samples were treated equally.

Reporting for specific materials, systems and methods

We require information from authors about some types of materials, experimental systems and methods used in many studies. Here, indicate whether each material, system or method listed is relevant to your study. If you are not sure if a list item applies to your research, read the appropriate section before selecting a response.

Materials & experimental systems

n/a	Involved in the study	
[ ]	[x]	Antibodies
[x]	[ ]	Eukaryotic cell lines
[x]	[ ]	Palaeontology and archaeology
[ ]	[x]	Animals and other organisms
[ ]	[x]	Human research participants
[x]	[ ]	Clinical data
[x]	[ ]	Dual use research of concern

Methods

n/a	Involved in the study	
[x]	[ ]	ChIP-seq
[ ]	[x]	Flow cytometry
[x]	[ ]	MRI-based neuroimaging

Antibodies

Antibodies used	Antibodies (ThermoFisher) against CD45 (CD45 Monoclonal Antibody (30-F11), PE-Cyanine5, eBioscience™ Cat#: 15-0451), Ter119 (TER-119 Monoclonal Antibody (TER-119), PE-Cyanine5, eBioscience™ Cat#: 15-5921), CD51 (CD51 (Integrin alpha V) Monoclonal Antibody (RMV-7), PE, eBioscience™ Cat#: 12-0512), Tie2 (CD202b (TIE2) Monoclonal Antibody (TEK4), eBioscience™ Cat#14-5987), Thy1.1 (CD90.1 (Thy-1.1) Monoclonal Antibody (HIS51), APC-eFluor™ 780, eBioscience™ Cat#: 47-0900), Thy1.2 (CD90.2 (Thy-1.2) Monoclonal Antibody (53-2.1), APC-eFluor™ 780, eBioscience™ Cat#: 47-0902), 6C3 (CD249 (BP-1) Monoclonal Antibody (6C3), APC,

	eBioscience™ Cat#: 17-5891), CD105 (CD105 (Endoglin) Monoclonal Antibody (MJ7/18), Biotin, eBioscience™ Cat#: 13-1051), and Streptavidin-PE-Cy7 conjugate (eBioscience™ Streptavidin PE-Cyanine7 Conjugate Cat#: 25-4317-82), Sca1 (Pacific Blue™ anti-mouse Ly-6A/E (Sca-1) Antibody Biolegend; Cat#: 108120). CD45 (Biolegend, Pacific Blue™ anti-human CD45 Antibody, HI30, Cat#30409,), CD235(Biolegend, Pacific Blue™ anti-human CD235ab Antibody, HIR2, Cat#306612,), CD31 (Invitrogen,CD31 (PECAM-1) Monoclonal Antibody (WM-59 (WM59)), Biotin, eBioscience™ Cat#13-0319-82), CD202b (Biolegend, Biotin anti-human CD202b (Tie2/Tek) Antibody, 33.1(Ab33), Cat#334204), CD146 (Biolegend, PE/Cyanine7 anti-human CD146 (MUC18, Mel-CAM) Antibody, SHM-57, Cat#342010), Podoplanin (Invitrogen, Podoplanin Monoclonal Antibody (NZ-1.3), APC, eBioscience™, Cat#17-9381-42), CD164 (Biolegend, PE anti-human CD164 Antibody, 67D2, Cat#324808), CD73 (Biolegend, FITC anti-human CD73 (Ecto-5'-nucleotidase) Antibody, AD2,Cat#344016)
Validation	Antibodies were validated according to manufacture's description.<br>CD45(Cat#: 15-0451):https://www.thermofisher.com/antibody/product/CD45-Antibody-clone-30-F11-Monoclonal/15-0451-82. Ter119 (Cat#: 15-5921): https://www.thermofisher.com/antibody/product/TER-119-Antibody-clone-TER-119-Monoclonal/15-5921-82. CD51 (Cat#: 12-0512): https://www.thermofisher.com/antibody/product/CD51-Integrin-alpha-V-Antibody-clone-RMV-7-Monoclonal/12-0512-82, Tie2 (14-5987): https://www.thermofisher.com/antibody/product/CD202b-TIE2-Antibody-clone-TEK4-Monoclonal/14-5987-82 Thy1.1 (Cat#: 47-0900) https://www.thermofisher.com/antibody/product/CD90-1-Thy-1-1-Antibody-clone-HIS51-Monoclonal/14-0900-81. Thy1.2 (Cat#: 47-0902) https://www.thermofisher.com/antibody/product/CD90-2-Thy-1-2-Antibody-clone-53-2-1-Monoclonal/47-0902-82 6C3 (Cat#: 17-5891) https://www.thermofisher.com/antibody/product/CD90-2-Thy-1-2-Antibody-clone-53-2-1-Monoclonal/47-0902-82 CD105 (Cat#: 13-1051) https://www.thermofisher.com/antibody/product/CD105-Endoglin-Antibody-clone-MJ7-18-Monoclonal/13-1051-82 Streptavidin-PE-Cy7 conjugate (Cat#: 25-4317-82) https://www.thermofisher.com/order/catalog/product/25-4317-82 Sca1 (Biolegend; Cat#: 108120) https://www.biolegend.com/it-it/products/pacific-blue-anti-mouse-ly-6a-e-sca-1-antibody-3140 CD45 (Biolegend, Pacific Blue™ anti-human CD45 Antibody, HI30, Cat#30409, https://www.biolegend.com/it-it/products/pacific-blue-anti-human-cd45-antibody-3331), CD235 (Biolegend, Pacific Blue™ anti-human CD235ab Antibody, HIR2, Cat#306612, https://www.biolegend.com/it-it/products/pacific-blue-anti-human-cd235ab-antibody-6242), CD31 (Invitrogen,CD31 (PECAM-1) Monoclonal Antibody (WM-59 (WM59)), Biotin, eBioscience™ Cat#13-0319-82, https://www.thermofisher.com/antibody/product/CD31-PECAM-1-Antibody-clone-WM-59-WM59-Monoclonal/13-0319-82), CD202b (Biolegend, Biotin anti-human CD202b (Tie2/Tek) Antibody, 33.1(Ab33), Cat#334204, https://www.biolegend.com/it-it/products/biotin-anti-human-cd202b-tie2-tek-antibody-4910), CD146 (Biolegend, PE/Cyanine7 anti-human CD146 (MUC18, Mel-CAM) Antibody, SHM-57, Cat#342010, https://www.biolegend.com/it-it/products/pe-cyanine7-anti-human-cd146-muc18-mel-cam-antibody-6939), Podoplanin (Invitrogen, Podoplanin Monoclonal Antibody (NZ-1.3), APC, eBioscience™, Cat#17-9381-42, https://www.thermofisher.com/antibody/product/Podoplanin-Antibody-clone-NZ-1-3-Monoclonal/17-9381-42), CD164 (Biolegend, PE anti-human CD164 Antibody, 67D2, Cat#324808, https://www.biolegend.com/it-it/products/pe-anti-human-cd164-antibody-3806), CD73 (Biolegend, FITC anti-human CD73 (Ecto-5'-nucleotidase) Antibody, AD2,Cat#344016, https://www.biolegend.com/it-it/products/fitc-anti-human-cd73-ecto-5-nucleotidase-antibody-8356)

Animals and other organisms

Policy information about studies involving animals; ARRIVE guidelines recommended for reporting animal research

Laboratory animals	C57BL/6 mice were purchased from Charles River. The ESR2 Knock-out mouse strain, B6.129P2-Esr2tm1Unc/J were purchased from Jackson Lab. All mice were housed in temperatures of 65-75°F with 40-60% humidity with a 14-hour light/10-hour dark cycle, and water is accessible at all times according to the protocols. The animals used in this experiment were 8 week old female mice. C57BL/6 aged female mice were operated on at 26 months old. 14 week old male were used in this study to account for the defined latent period in female mice bone mass/volume experiments (8 week old female mice + 6 weeks after ovariectomy).
Wild animals	This study did not involve wild animals.
Field-collected samples	This study did not involve samples collected from the field.
Ethics oversight	The study protocol was approved by Stanford’s Administrative Panel on Laboratory Animal Care.

Note that full information on the approval of the study protocol must also be provided in the manuscript.

Human research participants

Policy information about studies involving human research participants

Population characteristics	Regarding the adult tissue specimens, the typical patient is a middle- to advanced-aged man or woman. The tissues donated should be representative of the patient population seen in the Stanford University Hospital and Clinics. Based upon the demographics of the patients seen by the Orthopaedic Surgery Department at Stanford University, we anticipate that approximately 60% of the persons who donate tissue will be Caucasian, 15% Asian-Americans 10% Hispanic, 10% African-American, and 5% Pacific Islander/Alaska Native/American Indian. Of these patients, we anticipate approximately 50% will be female and 50% will be male. While the age of patients undergoing such procedures can range from 20-80+ years old, the majority of patients fall between 50 and 79.We anticipate a smaller number of tissue specimens from children aged 0 to 17 years in rare cases of high impact bone traumas requiring major surgery with a similar demographic distribution as for the adults. Fetal specimens used in this study will be approximately 17 weeks post-gestation.
Recruitment	No specific exclusion criteria will be employed during this study with the exception of specimens deemed unsafe (e.g. infection) or unfit (e.g. tissue damage) for extensive analysis.
Ethics oversight	The protocol is approved by the Institutional review boards(IRBs) at Stanford, which is also formally known as Administrative Panels for the Protection of Human Subjects. Fractured long bone specimens (20–90 years old) were obtained from Stanford Hospital in accordance with guidelines set by the Institutional Review Board(IRB-35711).The tissues utilized in this research

do not require consent per our IRB Protocol # 35711 and are considered exempted specimens per FDA guidelines 45 CFR 46.116 and therefore do not require consent forms.

Note that full information on the approval of the study protocol must also be provided in the manuscript.

Flow Cytometry

Plots

Confirm that:

- [x] The axis labels state the marker and fluorochrome used (e.g. CD4-FITC).
- [x] The axis scales are clearly visible. Include numbers along axes only for bottom left plot of group (a 'group' is an analysis of identical markers).
- [x] All plots are contour plots with outliers or pseudocolor plots.
- [x] A numerical value for number of cells or percentage (with statistics) is provided.

Methodology

Sample preparation

Isolation of mouse skeletal progenitor cells
Fractured and uninjured femoral shafts were harvested at post-fracture day 7. Mouse skeletal progenitors were isolated as described by Gulati et al.66 In brief, fracture calluses were dissected and finely crushed using pestle and mortar. Tissues underwent serial enzymatic digestion in collagenase buffer [collagenase 2.2mg/ml, deoxyribonuclease, IM CaCl2 (1000x), p188 (100X), 1M HEPES (50X), Medium 199] at 37oC for 30 minutes under gentle agitation. Dissociated cells were filtered through a 40um nylon mesh and washed with FACS buffer [PBS, 2% fetal bovine serum, 1% pen strep, 1% p188]. Cells were pelleted at 200G at 4oC and resuspended in FACS buffer. The suspension was layered onto a Histopaque gradient and centrifuged at 500G for 15 minutes at room temperature with zero acceleration. The cloudy cellular interphase was aspirated, washed with FACS buffer, and centrifuged. The cells were stained with fluorochrome-conjugated antibodies against CD45 at dilution of 1:200(Biolegend, San Diego, CA, USA), Tie2 at dilution of 1:20 (eBioscience, San Diego, CA, USA), AlphaV integrin at dilution of 1:50 (eBioscience), CD105 at dilution of 1:50 (eBioscience), Thy1.1 at dilution of 1:100 (eBioscience), Thy 1.2 at dilution of 1: 100 (eBioscience), 6C3 at dilution of 1:100 (Biolegend), and CD200 at dilution of 1:50 (Biolegend) for fractionation by fluorescence-activated cell sorting (BD FACSAria II in the Shared FACS Facility in the Lokey Stem Cell Institute).

Isolation of human bone progenitors
Femoral heads were obtained from young males, elderly males, premenopausal and postmenopausal females undergoing total hip arthroplasty. The bone marrow of the femoral head was isolated and human skeletal cells were separated from RBCs and bone dust by density gradient separation using 1:1 Histopaque 1.119 g/mL. The buffy coat was collected, washed with staining media, and the resulting cell suspension was depleted of CD45+ cells by magnetic-activated cell sorting (MACS). Cells were blocked with mouse IgG and stained with fluorochrome-conjugated antibodies against CD45 (Biolegend), CD235 (Biolegend), CD31 (eBioscience), CD202b (Biolegend), CD146 (Biolegend), Podoplanin (Biolegend), CD164 (Biolegend), CD73 (Biolegend). Flow cytometry was performed on FACS Aria II in the shared FACS Facility in the Lorry I. Lokey Stem Cell Institute. Gating schemes were established with fluorescence-minus-one (FMO) controls and 4',6-diamidino-2-phenylindole (DAPI) was used for viability staining.

Instrument

Flow cytometry was performed on FACS Aria II (BD Biosciences).
Gating schemes were established with fluorescence-minus-one (FMO: staining with all fluorophores except one) controls and negative propidium iodide (PI) (Sigma-Aldrich, Cat#P4170) staining (1 mg/ml) was used as a measure for cell viability.

Software

FlowJo 10 was used to analyze FACS data.

Cell population abundance

Quantification of cell populations are provided as a total number of percentages as described in the manuscript.Gating schemes were established with fluorescence-minus-one (FMO) controls, negative propidium iodide (PI) (Sigma-Aldrich, Cat#P4170) staining (1 mg/ml) was used as a measure for cell viability. Targeted population negative for PI staining were sorted using purity sort mode on FACS Aria II.

Gating strategy

Gating schemes were established with fluorescence-minus-one (FMO) controls

- [x] Tick this box to confirm that a figure exemplifying the gating strategy is provided in the Supplementary Information.
